# Automated assessment of human engineered heart tissues using deep learning and template matching for segmentation and tracking

**DOI:** 10.1002/btm2.10513

**Published:** 2023-04-18

**Authors:** José M. Rivera‐Arbeláez, Danjel Keekstra, Carla Cofiño‐Fabres, Tom Boonen, Milica Dostanic, Simone A. ten Den, Kim Vermeul, Massimo Mastrangeli, Albert van den Berg, Loes I. Segerink, Marcelo C. Ribeiro, Nicola Strisciuglio, Robert Passier

**Affiliations:** ^1^ Department of Applied Stem Cell Technologies, TechMed Centre University of Twente Enschede the Netherlands; ^2^ BIOS Lab on a Chip Group, MESA+ Institute for Nanotechnology, TechMed Centre, Max Planck Institute for Complex Fluid Dynamics University of Twente Enschede the Netherlands; ^3^ Data Management & Biometrics (DMB) Group University of Twente Enschede the Netherlands; ^4^ River BioMedics Enschede the Netherlands; ^5^ Microelectronics, TU Delft Delft the Netherlands; ^6^ Department of Anatomy and Embryology Leiden University Medical Centre Leiden the Netherlands

**Keywords:** automated tracking, cardiac performance, contractile force, deep learning, engineered heart tissues, segmentation, sub‐pixel interpolation, template matching

## Abstract

The high rate of drug withdrawal from the market due to cardiovascular toxicity or lack of efficacy, the economic burden, and extremely long time before a compound reaches the market, have increased the relevance of human in vitro models like human (patient‐derived) pluripotent stem cell (hPSC)‐derived engineered heart tissues (EHTs) for the evaluation of the efficacy and toxicity of compounds at the early phase in the drug development pipeline. Consequently, the EHT contractile properties are highly relevant parameters for the analysis of cardiotoxicity, disease phenotype, and longitudinal measurements of cardiac function over time. In this study, we developed and validated the software HAARTA (Highly Accurate, Automatic and Robust Tracking Algorithm), which automatically analyzes contractile properties of EHTs by segmenting and tracking brightfield videos, using deep learning and template matching with sub‐pixel precision. We demonstrate the robustness, accuracy, and computational efficiency of the software by comparing it to the state‐of‐the‐art method (MUSCLEMOTION), and by testing it with a data set of EHTs from three different hPSC lines. HAARTA will facilitate standardized analysis of contractile properties of EHTs, which will be beneficial for in vitro drug screening and longitudinal measurements of cardiac function.

## 1 INTRODUCTION

The global drug discovery market is estimated to be 74.96 billion dollars in 2021, and it takes on average 12 years before a new drug compound reaches the patients.^1^, ^2^, ^3^ From those compounds that reached the market, only very few offer important clinical advantages for the patients.^4^ Cardiotoxicity is what claims the highest incident of adverse drug reaction during preclinical or clinical development and post‐approval stage.^5^, ^6^ As a consequence, approximately one‐third of all drugs are withdrawn from the market.^5^, ^7^, ^8^ One of the reasons for this low success rate is the use of animal models as a preclinical method to evaluate efficacy and predict cardiotoxicity, which lacks high throughput and is expensive. Furthermore, animal models do not fully recapitulate human physiology and disease patho‐physiology.^9^ Currently, cardiomyocytes (CMs) can be obtained by differentiation of human pluripotent stem cells (hPSCs).^10^, ^11^ It has been previously shown that three‐dimensional (3D) hPSC‐derived cardiac models have several advantages over two‐dimensional (2D) cardiac in vitro models, because of their higher resemblance of the in vivo situation, based on improved molecular, morphological, and functional analysis. Standardized and automated analysis of cardiac function in 3D cardiac in vitro models will facilitate and expedite drug discovery and toxicity screening.^2^, ^12^, ^13^, ^14^ Using either control or patient‐derived hPSCs, 3D engineered heart tissues (EHTs) have been frequently used for studying cardiac function. In addition, EHTs are suitable for evaluation of pharmacodynamics and pharmacokinetics during the process of drug development.^12^, ^15^, ^16^, ^17^ We have previously developed a platform that allows to produce EHTs in a 12 well‐plate format.^18^ In this platform, the EHTs from hPSC‐CMs are formed around two cantilevers and there is a set of three pairs of cantilevers per well. Like this platform, multiple platforms use image analysis as a way to measure the contractility of the EHTs.^12^, ^15^, ^19^, ^20^, ^21^

The contractile properties of EHTs are relevant parameters for the analysis of disease phenotype, cardiotoxicity, and longitudinal measurements of cardiac function over time. Currently, there is a lack of a robust software that can analyze the contractile performance in a reliable and high throughput manner. One of the reference software for contractile analysis is MUSCLEMOTION.^22^ It uses dynamic changes in pixel intensity between video frames and creates a relative measure of movement as output, which means that there are no absolute values of force, making it difficult to compare functional properties between tissues. Besides, the accuracy of this method suffers from distortions, problems in case of background noise, sample shifts, vibrations, and susceptibility to the presence of non‐desired objects (e.g., debris) in the videos. These problems limit the algorithm robustness and the usability on a large data set. Meanwhile, the field of computer vision has evolved rapidly, with the development of methods based on deep (neural network) learning and template matching, which are used to speed up and increase the accuracy of analyzing biological samples.^23^, ^24^, ^25^, ^26^, ^27^ Deep learning algorithms are used, for instance, in image segmentation methods to classify each pixel of an image belonging to one of the regions of interest. State‐of‐the‐art approaches use convolutional networks that are trained using labeled examples.^28^ Template matching is a method for finding a region similar to a given template object in a source image. This is done by comparing a template image T to every possible region in a source image I using a sliding window approach.^29^ We hypothesize that by developing an algorithm that combines the methods of computer vision like deep learning and template matching for analysis of contractile parameter in EHTs, we will overcome the current limitations of the state‐of‐the‐art image analysis in MUSCLEMOTION, related to relative output values, the sample shifts, background noise, and susceptibility to the presence of artifacts. This will increase the accuracy of measurement and facilitate the automatic analysis of large data sets.

In this study, we developed HAARTA (Highly Accurate, Automatic and Robust Tracking Algorithm), a software tool that automatically analyzes contractile proprieties of EHTs by segmenting and tracking brightfield videos, using deep learning and template matching with sub‐pixel precision. We evaluated the software with a data set of hPSC‐derived EHTs brightfield videos and use it in following the treatment of the positive inotropic agent isoproterenol. We demonstrate improved robustness and accuracy of the newly developed software HAARTA by comparing it with MUSCLEMOTION, leading to standardized analysis of contractile properties of EHTs, which will be beneficial for in vitro drug screening and longitudinal measurements of cardiac function.

## 2 RESULTS

### 2.1 Overview

**FIGURE 1:**
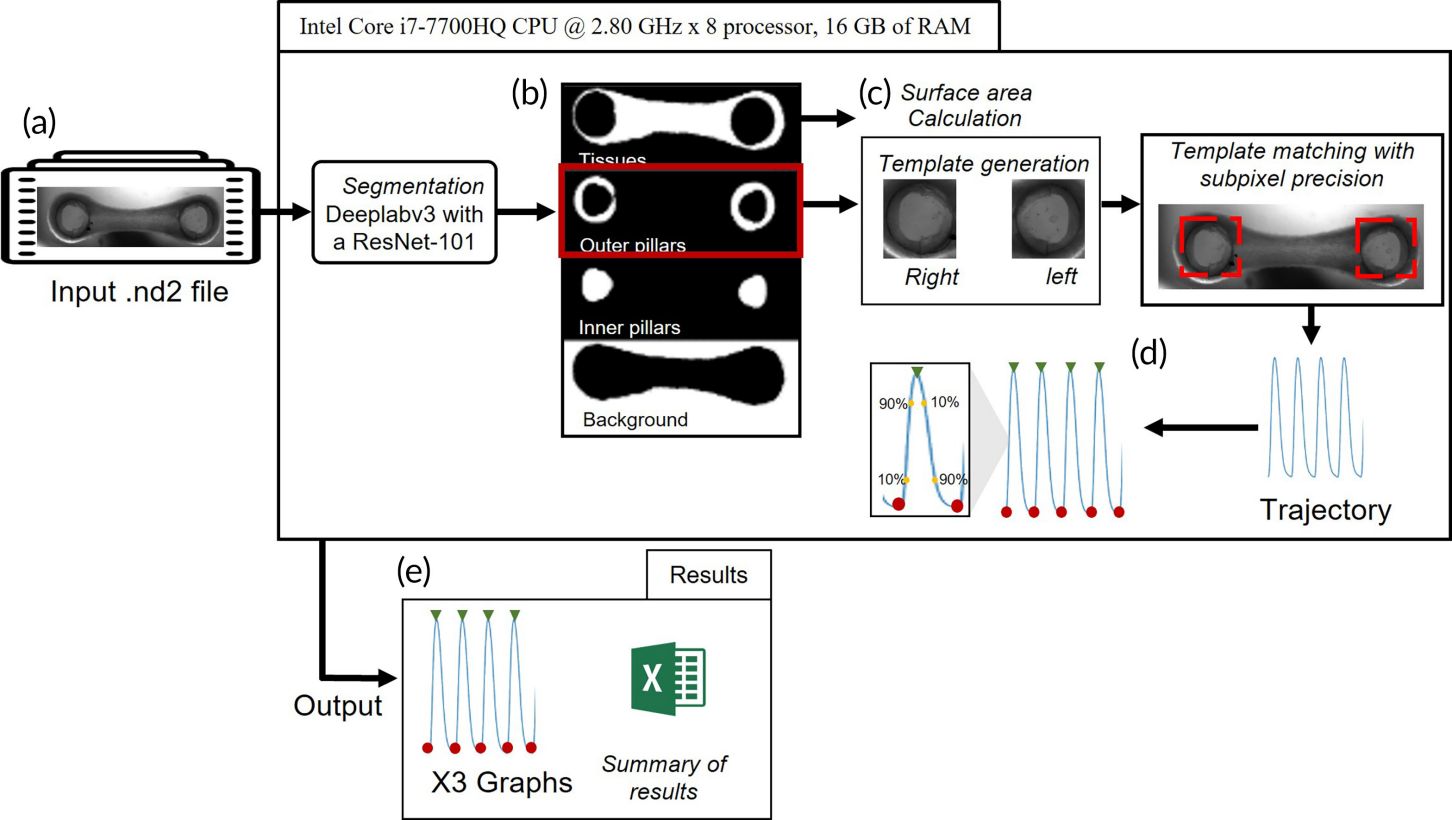
Workflow of the algorithm. (a) Input brightfield EHT video in ND2 format. (b) Four segmented templates after using Deeplabv3 with a ResNet‐101 backbone (tissue, outer part of the pillar, inner part of the pillar, and background). (c) Calculation of surface area with the tissue template and selection of the pillars template. (d) Calculation of contractile properties and contraction kinetics using the contraction trajectory after template matching with sub‐pixel precision. (e) Results folder with the raw data and contraction graphs. EHT, engineered heart tissue.

We developed HAARTA, a software that facilitates the automatic assessment of contractile properties of EHTs, which integrates deep learning and template matching techniques. HAARTA initially segments a brightfield frame of an EHT video using a pre‐trained Deeplabv3 with a ResNet‐101^30^ as convolutional backbone (Figure 1a). The algorithm segments the frame in four regions and identifies the 3D cardiac tissue, the outer pillar where the tissue is anchored to, the inner part of the pillar, and the background (Figure 1b). The algorithm uses first the segmented region of the tissue to calculate the surface area. Subsequently, the outer pillar region is used as a template for tracking the position of the pillars during tissue contraction (Figure 1c). We considerably increased the accuracy of the tracking by template matching with sub‐pixel precision, which allows to detect movements below pixel resolution. The trajectory result of the tracking is used to calculate maximum and minimum contraction, contraction kinetics, and the times that takes to achieve 10% and 90% of contraction and relaxation (Figure 1d). Graphs and raw data are located in a “Results” folder together with a summary file (Figure 1e). The key to our method is the combination of an accurate segmentation step, which allows having the right template to use in the tracking by template matching with sub‐pixel precision, which results in automatic, robust, and standardized tracking of EHT datasets.

### 2.2 Tracking by template matching with sub‐pixel precision

**FIGURE 2:**
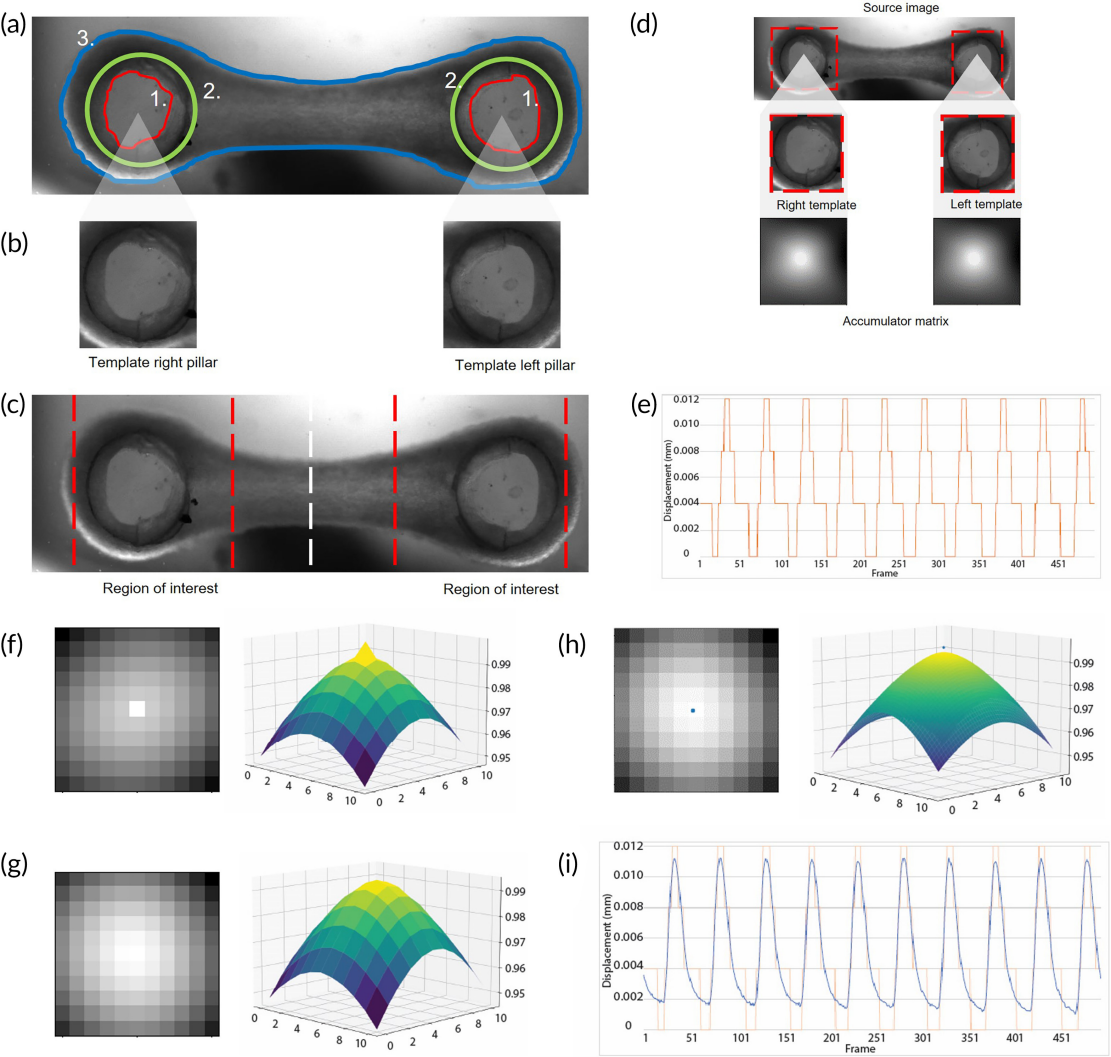
Brightfield of EHT around commercial pillars and template matching. (a) Segmented regions of interest; (a‐1) inner area of the pillar, (a‐2) area of the outer ring, and (a‐3) tissue surface area. (b) Template of the right and left pillar. (c) Frame division by region of interest. (d) Accumulator matrix of the template matching of the EHT. (e) Trajectory over time of an EHT tracked with template matching. (f) Result peak region image of template matching method exactly on pixel matched, and its corresponding 3D projection. (g) Result peak region image of the template matching method where the match lays between pixels and its corresponding 3D projection. (h) Result peak region image by the template matching algorithm with annotated sub‐pixel precision match and its corresponding interpolated 3D projection. (i) Trajectory of an EHT tracked by template matching with sub‐pixel precision. EHT, engineered heart tissue.

For contractile assessment of EHTs, tracking the centroid of the anchored points by using a thresholding algorithm is the most common technic in multiple platforms^31^, ^32^, ^33^, ^34^ (Table S1). In the case of the pillars provided by River BioMedics, the variability in the bottom shape and the low level of contrast in the brightfield videos, combined with background noise, make it challenging to use that approach (Figure S1). In order to overcome this limitation and obtain more robust and accurate tracking results, template matching was implemented. Initially, the regions of interest in an EHT video were identified. Those regions are defined as the background, 3D cardiac tissue, and the pillars. Specifically, the pillars were divided in outer pillar and inner pillar (Figure 2a). As a result of the variability observed in the shape of the inner pillar (Figure S1A), the outer region was initially chosen as template and manually segmented on the first frame successfully (Figure 2b). To have significant reduction of computational costs during the tracking by template matching, we took into account what has been shown previously about the behavior of 3D cardiac tissue during contraction, to define a region within the frame to look for displacement of the pillar^17^, ^34^, ^35^ (Figure 2c). Therefore, the template matching was computed only on those regions. As a result we obtained an accumulator matrix, wherein the brighter the pixel is, the better the match (Figure 2d). The contraction trajectory created by calculating the distances between the brighter points detected on the left and right pillars showed a square contraction wave (Figure 2e) (Equations 4 and 5). It is worthy to note that this is not the expected smooth contraction wave according to literature.^35^, ^36^, ^37^

Therefore, we designed a tracking method based on sub‐pixel precision matrix. We interpolate in between pixels of the accumulator matrix, specifically in the region of maximum intensity. We observed the result of the template matching and the corresponding 3D projection (Figure 2f). This resulted in a perfect match, shown by the peak in the 3D projection. However, this is not always the case, since occasionally the match lies between two pixels (Figure 2g). This is shown by neighboring pixels that have a match score close to the maximum score. To find the perfect match in this case we used interpolation and identified the match point of the template with sub‐pixel precision (Figure 2h). We used this method to identify the center of the pillars along the frames and obtained a smooth contraction wave (Figure 2i).

### 2.3 Segmentation by deep learning

**FIGURE 3:**
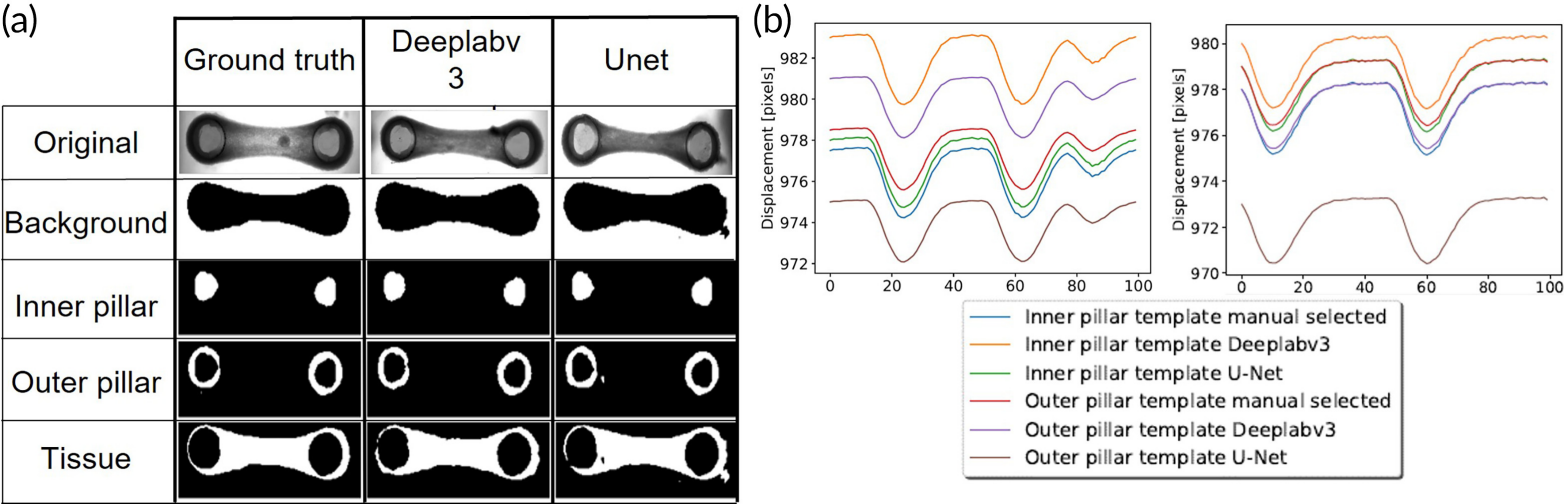
Segmentation by two convolutional neural networks (CNNs). (a) Segmentation ground truth, Deeplabv3 with a ResNet‐101 backbone prediction and U‐net prediction of the background, inner pillar, outer pillar, and tissue classes. (b) Comparison of the displacement over time computed using different templates. Specifically, inner and outer part of a pillar as template manually selected or by segmentation by U‐net and Deeplabv3.

For segmentation, deep learning approaches have been previously reported as valuable tool to identify image regions belonging to different objects of interest.^38^, ^39^, ^40^ In this study, we compared two convolutional neural networks (CNNs), namely Deeplabv3 with a ResNet‐101 backbone^30^ and U‐net.^40^ To train the networks, we manually annotated ground truth segmentation maps of 81 EHT brightfield videos (Figure S2). We used the annotated data to train and test the considered CNNs, and found that both approaches perform well in segmentation tasks of EHT regions. Nevertheless, U‐net showed slightly higher performance to identify the tissue, outer, and inner pillar (Table 1 and Figure 3a).

**TABLE 1:** Intersection over Union (IoU) results of the Deeplabv3 with a ResNet‐101 backbone and U‐net prediction of the background, inner pillar, outer pillar, and tissue classes.

Model	Outer (%)	Tissue (%)	Outer pillar (%)	Inner pillar (%)
Deeplabv3 ResNet‐101	96.7	96.0	90.1	91.2
U‐net	97.3	97.1	91.3	92.5

Towards an automatic assessment of the contractility of EHTs and eliminating the manual selection of the template described above, we evaluated the use of the two segmentation CNNs to provide input templates for the template matching algorithm. Accordingly, we identified that, regardless of the differences in trajectory baseline, the computed trajectories using automatically extracted templates are very similar. Overall, selecting the outer pillar templates contributes to having a smoother trajectory than the inner pillar template trajectories (Figure 3b).

### 2.4 Validation of the template matching with sub‐pixel precision using a simulation video

**FIGURE 4:**
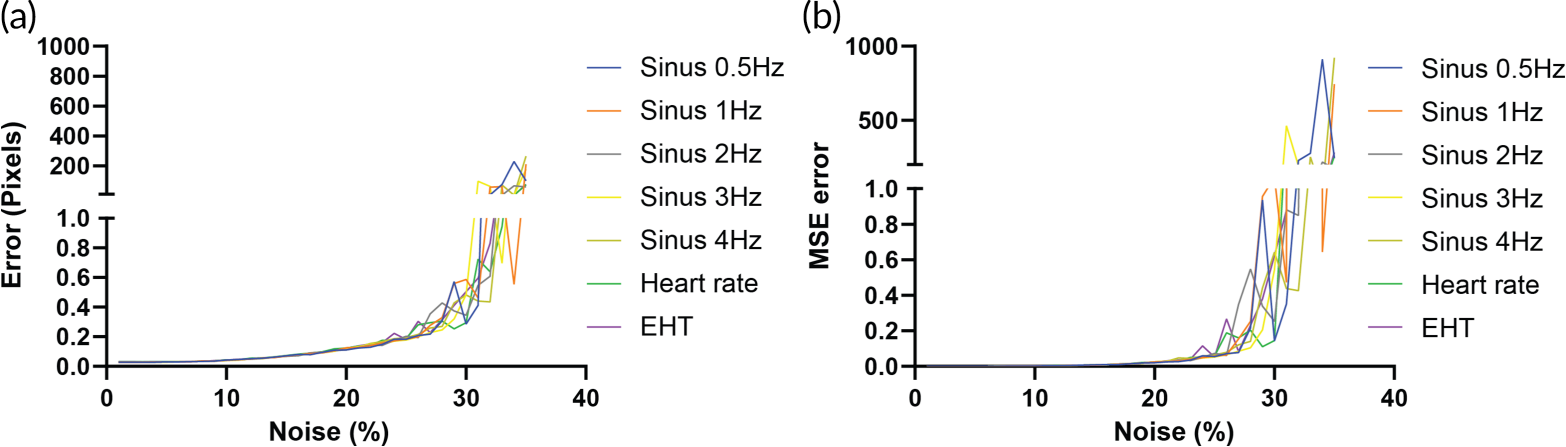
Template matching with sub‐pixel precision using a simulation video. (a) Mean absolute error results of the simulated videos of different signals tracked by the proposed template/pattern matching with sub‐pixel precision method compared to the ground truth. (b) Mean square error results of the simulated videos of different signals tracked by the proposed template/pattern matching with sub‐pixel precision method compared to the ground truth.

To assess the performance of the tracking and segmentation, we made simulation videos where we control the background noise and the frequency of contraction (Figure S4). We evaluated the accuracy of the tracking by increasing the noise at different frequencies. We observed that the algorithm is stable until 25% of added noise. At that level, the error in pixels is below 0.2 approximately. With low levels of noise, the algorithm performs with a precision lower than 0.1 pixel (Figure 4).

As a complementary step, we qualitatively evaluated the results of the tracking on a scale from 1 to 5, where 5 is the best score and 1 is the lowest. The evaluation showed that 98% (79 videos) scored 5 on correct tracking, followed by 1% that scored 4 or 3. In all the videos the trajectory was correct, the 2% below the 5 score were due to a misalignment of the points printed on top of the frame with the pillars.

#### 2.4.1 Comparison of the tracking accuracies of our method and MUSCLEMOTION

**FIGURE 5:**
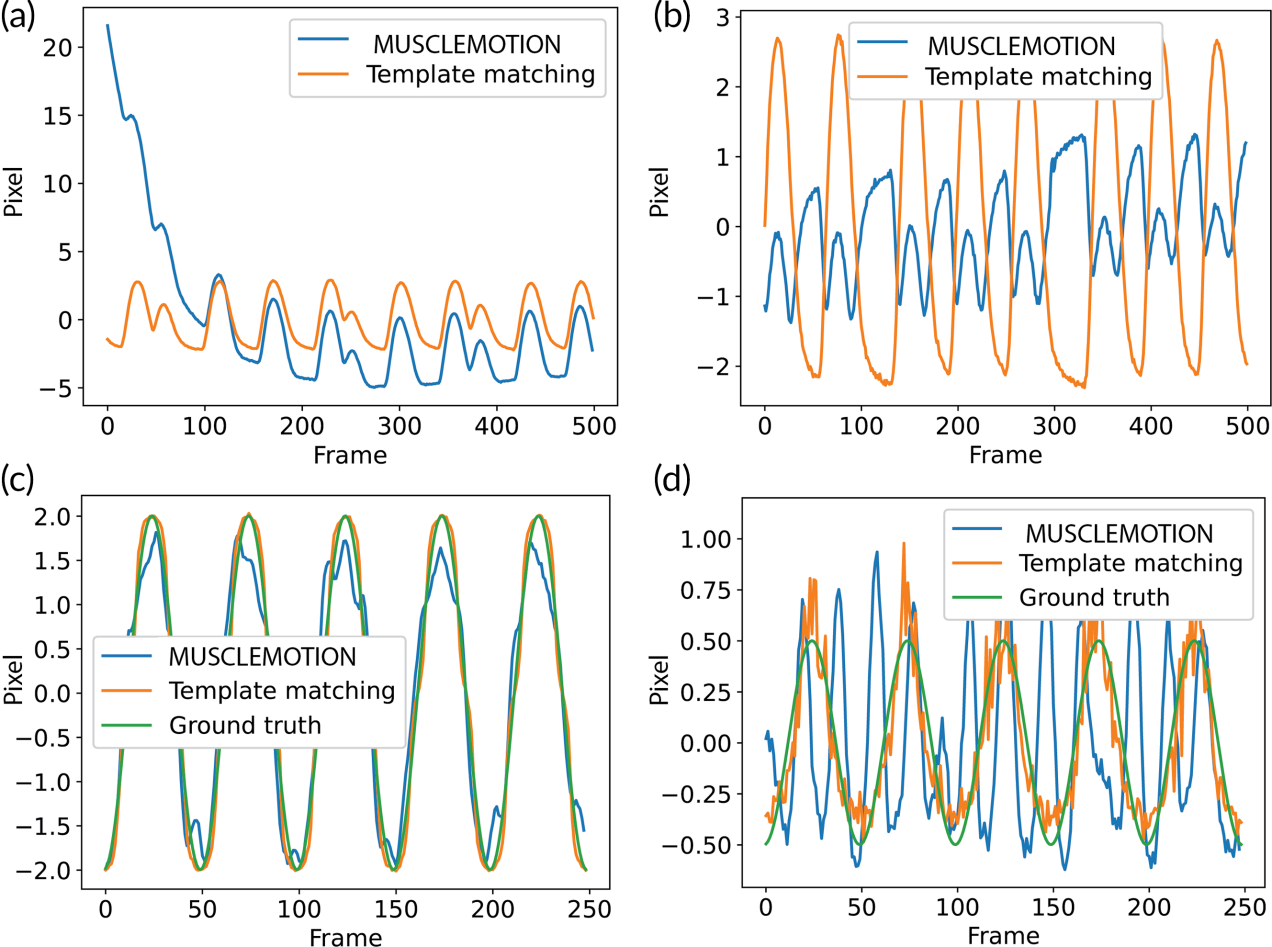
Comparison of tracking accuracy. (a and b) Comparison of a trajectory extracted from two separate EHT videos by MUSCLEMOTION (blue line) and by our template matching tracking with sub‐pixel precision (orange line). Comparison of a trajectory extracted from a simulation video of a 2 Hz sine with a 2‐pixel amplitude and 10% added noise (c), and 20% added noise (d). MUSCLEMOTION (blue line), template matching with sub‐pixel precision (orange line), and versus ground truth (green line). EHT, engineered heart tissue.

To further assess the potential of HAARTA, we compared the tracking performance with MUSCLEMOTION. We found that in multiple occasions our method outperformed the tracking accuracy of MUSCLEMOTION, and that only in situations where the displacement was significant (visible to the eye) and the background noise low, similar trajectories were observed (Figure 5a,b and Figure S5). Then, we compared the performance of both software with the ground truth and in the presence of different background noise levels. HAARTA consistently achieved higher accuracy in generating the contraction trajectories. In the case without background noise, the contraction trajectory computed by our algorithm was identical to the ground truth, while with MUSCLEMOTION some alterations in the trajectory were observed (Figure 5c,d and Figure S6).

### 2.5 Computational speed

The computation speed of the algorithms is measured. In our case, the computation speed of the proposed methods are measured on a high‐end desktop and a high‐end laptop (Table 2).

**TABLE 2:** Speed results of loading, segmentation, and template matching.

	Computer			
	Laptop		Desktop	
	CPU	GPU	CPU	GPU
	i7 6700HQ	GTX960m	i7 10700	RTX3080
	Time (s)			
Video loading	4.70	–	1.50	–
U‐Net model load	0.21	0.33	0.13	0.15
U‐Net segmentation	2.15	2.13	1.00	1.02
Deeplabv3 model load	2.31	5.34	1.19	2.19
Deeplabv3 segmentation	4.91	5.17	2.43	2.47
Inner pillar template matching	14.10	–	6.20	–
Outer pillar template matching	16.80	–	7.20	–

### 2.6 Contractile performance of human EHTs using a commercially available EHT platform

We tested the performance of the algorithm by analyzing the contractility of hPSC‐derived EHTs made using the 12‐well‐plate platform provided by River BioMedics. This platform has six pillars aligned in parallel. To have an accurate calculation of force of contraction, we determined the pillar stiffness by using a nanomechanical testing system and calculated the slope of the obtained force–displacement curve (Figure S7). The contractile force is consequently computed from the optically quantified displacement of the pillar tip induced by the tissue and the pillar stiffness. Subsequently, we successfully made EHTs using CMs differentiated from three different cell lines. Specifically, we used the human embryonic stem cell (hESC) NKX2.5^EGFP/+^‐COUP‐TFII^mCherry/+^ line, human induced pluripotent stem cell (hiPSC) line LUMC0020iCTRL‐06, and the commercially available iCell® from FUJIFILM Cellular Dynamics. We observed that the tissues formed and maintained spontaneously contractions over 30 days. First spontaneous contraction was visualized after 3 days of tissue formation in all cell lines and the contractile performance of the tissues was evaluated every 5 days. The hESC‐derived EHTs showed the highest contractile force at day 30 (0.6 mN) and a significant difference compared to day 5 was observed since day 20.

**FIGURE 6:**
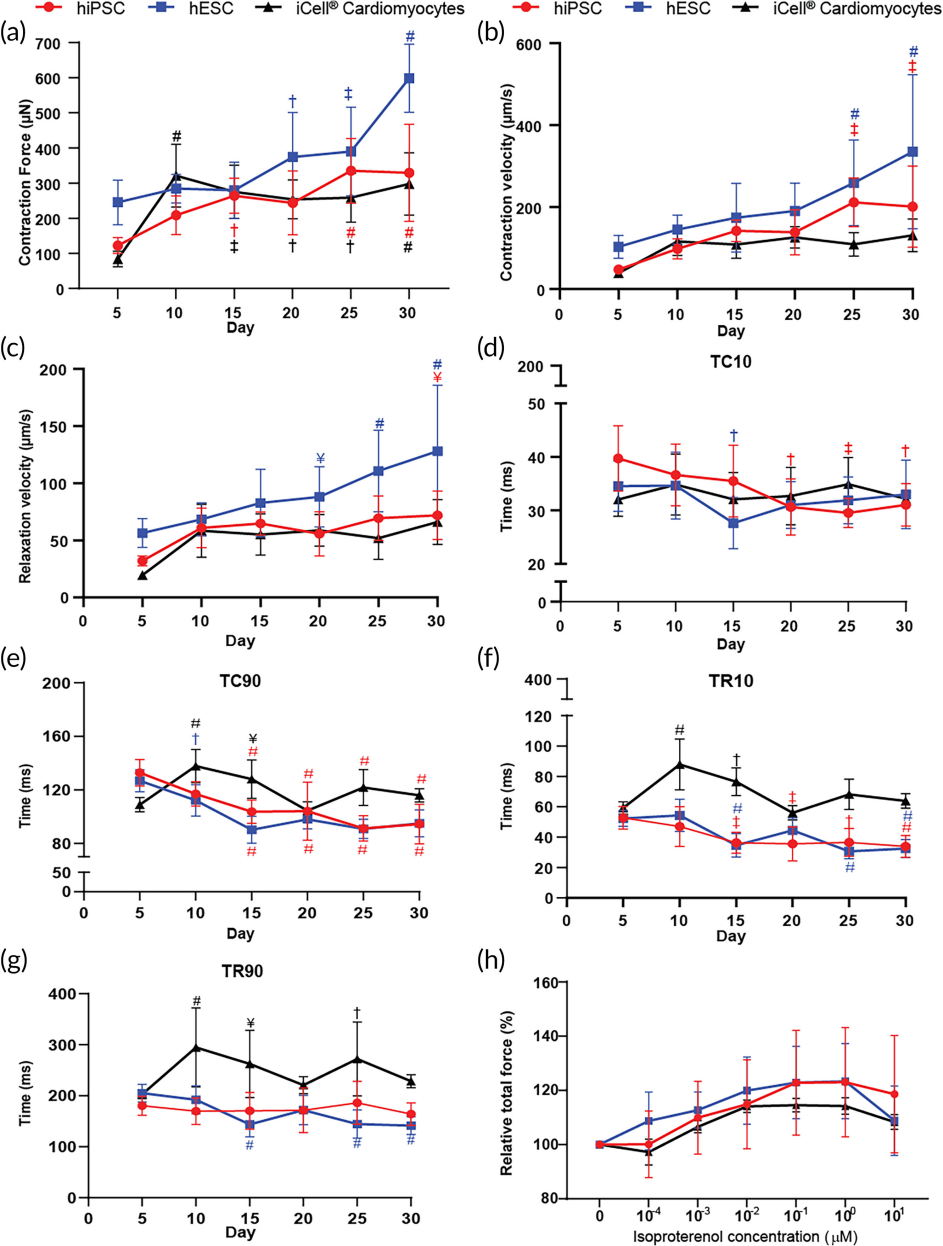
Contractile parameters of hPSC‐derived EHTs. (a) Contractile force of EHTs using three different cell lines at day 5, 10, 15, 20, 25, and 30. (b and c) Contraction velocity (b) and relaxation velocity (c) of EHTs at day 5, 10, 15, 20, 25, and 30. (d–g) Time to achieve 10% (TC10) (d) and 90% of contraction (TC90) (e); followed by the 10% (TR10) (f) and 90% (TR90) (g) of relaxation time at day 5, 10, 15, 20, 25, and 30. (h) Relative force response to inotropic agent isoproterenol (0–10 μM) in hESC, hiPSC, and iCell®‐EHTs. *p*‐values represented significant differences versus day 5 in each case and they are shown according to the color. Data are shown as means ± SD. Two‐way ANOVA plus Tukey's test for comparisons: ¥ = *p* < 0.05; † = *p* < 0.01; ‡ = *p* < 0.001; and # = *p* < 0.0001 (*N* = 3, biological replicates from independent differentiations). EHT, engineered heart tissue; hESC, human embryonic stem cell; hiPSC, human induced pluripotent stem cell; hPSC, human pluripotent stem cell.

In the case of hiPSC‐EHT, a significant higher contraction force was observed from day 15 to day 30 compared to the first measurement point (day 5). On the other hand, the iCell®‐EHTs had a considerable increase in force from day 5 to day 10 and afterwards the performance of the tissues was stable around 0.3 mN (Figure 6a). We observed a consistent increase in the velocity of contraction in all situations. Specifically, a significant increase in contraction velocity was observed at day 25 and 30 compared to day 5 in hiPSC‐EHTs and hESC‐EHTs. Differences in relaxation velocity were seen at day 20, 25, and 30 in hESC‐EHTS and only at day 30 in hiPSC‐EHTs (Figure 6b,c). Both contraction and relaxation velocity were calculated automatically by the algorithm. Accordingly, 10% and 90% of contraction times decreased over time in hiPSC‐EHTs and hESC‐EHTs. While the relaxation time was more stable over time, differences were observed from day 15 in hESC‐EHTs and hiPSC‐EHTs. Particularly in the case of iCell®‐EHTs, the time of contraction and relaxation was higher from day 5 to day 20 and afterward decreased in the last two time points (Figure 6e–g). The response to β‐adrenergic agonists, such as isoproterenol, is an important readout in EHTs,^19^, ^22^, ^41^ therefore we also tested the performance of the algorithm to analyze contractile parameters in tissues after treatment of a drug. Administration of isoproterenol from 0 to 10 μM led to an increase of approximately 20% in the relative force at 0.01 μM in all the conditions followed by a plateau phase. Also, at the highest concentration (10 μM) out of the normal physiological values, the software was able to analyze the EHTs contractility accurately without any problem. We observed a decrease in the relative force of the tissues at this concentration (Figure 6h).

## 3 DISCUSSION

The use of 3D cardiac tissues as an in vitro model for drug discovery, disease modeling, and longitudinal cardiac studies has increased over the last years.^17^, ^19^, ^20^, ^42^ However, a robust and accurate algorithm that allows easy and widespread contractile analysis, where background noise, undesired artifacts, or detection of subtle differences at the sub‐pixel level are not a problem, is still not available. Moreover, individual laboratories develop their own custom software (Table S1), using thresholding technics and for this, continuous optimization of multiple parameters is usually required. To solve this problem, we presented HAARTA which allows to automatically analyze contractile properties (contraction force, contraction kinetics, and contraction time) of EHTs. The algorithm is based on segmenting and tracking brightfield videos, using deep learning and template matching with sub‐pixel precision. We demonstrated the robustness, high‐performance, accuracy, and computational efficiency of the algorithm after analyzing multiple brightfield videos of different EHTs over time, and after treatment with drugs. Furthermore, we performed a quantitative and qualitative validation step using simulation videos. Notably, we showed that our algorithm successfully tracks at the sub‐pixel level with a 0.2 pixel precision with 25% of added image noise, and with a 0.1 pixel precision up to 20% of added noise. Moreover, to speed up the post‐analysis, the results from an analyzed data set are saved in a folder that contains the graphs (contraction force, contraction kinetics, and contraction time), raw data of each analyzed file, and a summary of all of them.

The robustness and high accuracy of our method is attributable to the use of a CNN in combination with the tracking by template matching with sub‐pixel precision. To our knowledge, this is the first time this has been performed for analysis of contractile properties of 3D cardiac tissues. Therefore, after generating the EHTs ground truth, defining the training data set, and assessing the performance of two deep learning models (U‐net and Deeplabv3 with resnet‐101 backbone), we found that the U‐net implementation scores the best in the Intersection over Union (IoU) test. Although U‐net scored the highest (Table 1), the visual results of the implementation showed extra segmented blobs in unexpected places (Figure 3a). These blobs may cause inaccuracies when selecting the template for the automatic tracking with template matching. For that reason, Deeplabv3 that scored slightly less than U‐net in terms of quantitative measures, is not impacted from the appearance of blobs, and is thus more appropriate to define the input template for the automatic tracking. The segmentation results of Deeplabv3 were above 0.9 IoU for all the classes (i.e., background, tissue, inner, and outer pillar) and the comparison of tracking results using different templates, showed similar trajectories (Figure 3b). However, as shown in Figure S1A, there is a high fabrication variability in the internal shape of the pillar while the outer shape is more regular. Thus, by using the Deeplabv3 to identify the outer pillar as an input for template matching with sub‐pixel precision, we obtained a more robust and accurate way to track the displacement of the pillars over time. It is important to note that U‐net is a deep learning model explicitly developed for biomedical image segmentation.^40^ Nevertheless, for this application of segmenting videos of 3D cardiac tissues with the pillars and using it as an input for template matching, we found that Deeplabv3 achieves better and more stable results.

There are several custom‐made algorithms that are used by other research groups (Table S1) to analyze contractility of cardiac tissues. In general, the thresholding technic and edge detection are the most used to identify and track displacement of pillars in brightfield videos.^20^, ^31^, ^33^, ^34^ However, this requires constant optimization and is susceptible to changes of illumination, background noise, and artifacts (e.g., debris), which is a problem for high‐throughput analysis. In the particular case of EHT technologies,^41^ a customized software package was developed by a private company that focus on the top and bottom of the tissue, which makes it specific for their platform and not easy to access. We compared the performance of our algorithm with that of MUSCLEMOTION,^22^ a software currently used in multiple laboratories to analyze contractility. This software focuses on the differences in pixel intensity between a references frame and the frame of interest to assess displacement. The results are thus sensitive to the correct selection of the reference frame and noise levels. As an output, it only gives relative values and not absolute values of contractility, which makes it difficult to compare the performance among tissues. We have shown that our algorithm overcomes the problem of thresholding, background noise, artifacts, and outperforms MUSCLEMOTION in the analysis of different brightfield videos of 3D cardiac tissues by accurately tracking and providing as an output a smooth contractile trajectory (Figure 5 and Figure S5). Additionally, our algorithm consistently outperforms MUSCLEMOTION when compared to the ground truth and in the presence of different noise levels (Figure S6). As indicated HAARTA is developed for analysis of pillar‐based contractile tissues. For other 3D or 2D configurations MUSCLEMOTION is a valuable tool for analysis of contraction parameters.

Nevertheless, a great addition to our algorithm would be to measure the level of noise present in a video before the analysis. This will improve the reliability of our algorithm by estimating the precision of the output trajectory with the found results of the simulation. In situations where a very low displacement is identified (in case of diseased or severely affected cardiac tissues), a low pass filter on the trajectories could be applied to avoid an increase in the relative error.

We evaluated the performance of the algorithm by analyzing the hPSC‐derived EHTs made using the 12‐well plate platform provided by River BioMedics with three different cell lines. We observed that the contractile force in all three cases is higher compared with previous results using polydimethylsiloxane (PDMS) pillars.^35^ This is related to the significant increase in the stiffness of the commercial pillars compared to PDMS, which creates a higher load to the tissues. As previously reported, the CMs increase the contractile performance when they are under higher mechanical restriction.^43^ Overall, EHTs from the different cell lines, started showing a significant higher contractile force and contraction kinetics between day 15 and day 20, and the highest performance was achieved at day 30. Additionally, in all experiments we observed an increase in contractile force in the presence of positive inotropic β‐adrenoceptor agonist isoproterenol, indicating proper performance of CMs.^19^, ^44^

In summary, we have demonstrated the advantages of HAARTA to analyze contractile properties of 3D cardiac tissues by using a new approach that combines segmentation and template matching with sub‐pixel precision, under different conditions. Furthermore, our algorithm successfully tracks at the sub‐pixel level with a 0.2 pixel precision up to 25% of added noise and with a 0.1 pixel precision up to 20% of added noise. We believe that the logic of the template matching with sub‐pixel precision will be very advantageous for the automatic and standardized analysis of other (commercial) platforms that are assessing contractile performance of engineered heart or muscle tissues.

## 4 CONCLUSION

To conclude, we present HAARTA, a robust, accurate, and computationally efficient algorithm for analyzing hallmark physiological features of brightfield videos of 3D cardiac tissues. After quantitative and qualitative evaluation, the software has shown to offer an advantage to the current methods to measure contractility. Moreover, with the standardization and miniaturization of contractile tissues (either cardiac or skeletal muscle), which can be generated from patient‐derived stem cells, the use of software for automatic and accurate functional analysis will be important for processing big data sets and will facilitate disease modeling and safety pharmacology and expedite drug discovery.

## 5 MATERIALS AND METHODS

### 5.1 Computational environment

Our assessment of the EHTs was performed on a personal computer with an Intel Core i7‐7700HQ CPU @ 2.80 GHz x 8 processor, 16 GB of RAM, and Microsoft Windows 10 Pro. We trained the neural networks with an NVIDIA 3080 RTX (10 GB). The manual labeling was done using Adobe Photoshop (RRID:SCR_014199).

### 5.2 Tracking

#### 5.2.1 Template matching

Template matching was used to track the position over time of the pillars by comparing the template image (*T*) of each set of two pillars extracted in the first frame through the stack of frames (source images (*I*)). This was done by comparing the pixel intensities with the normalized correlation coefficient (NCC).^29^

The corresponding formulas for NCC matching are found in Equation (1), Equation (2), and Equation (3). NCC computes an accumulator matrix *R*. Each value in the accumulator matrix represents a match score of the location in the source image where one indicates an exact match, and zero indicates no match at all. The location of the best match was found by determining the highest score in the matrix.

(1) $$R\left(x,y\right)=\frac{\sum_{x\prime ,y\prime }\left(T^{\prime }\left(x^{\prime },y^{\prime }\right)*I^{\prime }\left(x+x^{\prime },y+y^{\prime }\right)\right)}{\sqrt{\sum_{x\prime ,y\prime }T\prime {\left(x\prime ,y\prime \right)}^{2}*\sum_{x\prime ,y\prime }I^{\prime }{\left(x+x\prime ,y+y\prime \right)}^{2}}},$$

(2) $$T^{\prime }\left(x^{\prime },y^{\prime }\right)=I\left(x^{\prime },y^{\prime }\right)-\frac{\sum_{x^{\prime \prime },y^{\prime \prime }}\left(T^{\prime \prime }\left(x^{\prime \prime },y^{\prime \prime }\right)\right)}{\left(w-h\right)\;},$$

(3) $$I^{\prime }\left(x+x^{\prime },y+y^{\prime }\right)=I\left(x+x^{\prime },y+y^{\prime }\right)-\frac{\sum_{x\prime \prime ,y\prime \prime }\left(I\left(x^{\prime \prime },y^{\prime \prime }\right)\right)\;}{\left(w-h\right)}.$$

From the original frame, a region of interest was used to initialize the template matching algorithm for each pillar, taking into account the biological behavior of the cardiac tissues described in literature (Figure 2c). The brighter pixel from the accumulator matrix of the left and right matched pillar templates were used to calculate pixel distance between the points according to Equation (4). In this way, we tracked the distance of the pillar centroids frame‐by‐frame. Later, that distance was converted from pixel to millimeters (mm) and subtracted from the initial distance between the pillars to calculate the displacement per frame (Equation 5).

(4) $$T_{\text{pixel}}=\sqrt{{\left(X_{\text{left}}-X_{\text{right}}\right)}^{2}+{\left(Y_{\text{left}}-Y_{\text{right}}\right)}^{2}},$$

(5) $$T=T_{\text{pixel}}*a-3.2\;\mathrm{mm},$$

where a is the conversion factor from pixels to millimeters and 3.2 mm, the initial distance between the pillars.

#### 5.2.2 Tracking using sub‐pixel precision

We used the output of the NCC accumulator matrix to identify with higher precision the displacement of the pillars as the movement is below pixel level resolution. We zoomed the region of the maximum intensity to find a match with sub‐pixel precision by using spatial interpolation methods. Using a polynomial function, we estimated the values between pixels and used the pixel with higher match score. Bicubic spline is a common technique for obtaining smoothness in two‐dimensional interpolation.^45^ By using the function output of the Bicubic spline we got a match score for a (decimal) location in the peak region and beyond. To find the highest match score we used the local maximum of the function by using the Nelder–Mead algorithm.^46^ The Nelder–Mead algorithm is one of the best‐known algorithms for multidimensional unconstrained optimization without derivatives. The location of the maximum match score at pixel level is already known and was the starting point to find the local maximum of the function. The initial point is within half a pixel distance of the maximum match score, and therefore, the computation of the Nelder–Mead algorithm is limited together with a threshold (we set it to 0.0001 pixel). Using the output of this method the trajectory of the contraction at sub‐pixel precision was determined.

### 5.3 Verification step

To verify the performance of the tracking, simulation videos were made. First, a video was recorded with the same setting of an experiment but without any tissue, so to inspect the baseline noise level, illumination, and magnification. On top of the background video, a simulation of a moving tissue was created. We used a segmented tissue, cut it in half and placed it on top of the background. To simulate the movement with sub‐pixel precision, an affine transformation was used to translate the two parts to a sub‐pixel precise location over the video. Additionally, to recreate a normal scenario and test the robustness of the tracking technic, random noise of increasing energy was added to the simulation video. To measure the error from the ground truth, the mean absolute error (MAE) (Equation 6) and mean square error (MSE) were calculated (Equation 7):

(6) $$\mathrm{MAE}=\frac{1}{n}\sum_{i=1}^{n}\left(y_{i}-x_{i}\right),$$

(7) $$\mathrm{MSE}=\frac{1}{n}\sum_{i=1}^{n}{\left(y_{i}-x_{i}\right)}^{2}.$$

### 5.4 Segmentation using deep learning

#### 5.4.1 Data set

In this study, a data set of manually segmented EHT frames was created to provide a CNN with ground truth data of the EHT brightfield's. A set of 65 videos of approximately 500 brightfield frames per video was used to create the data set. Four frames per video were manually segmented, those frames were at the positions 0, 150, 300, and 450. The dataset has 260 brightfield images with their corresponding pixel‐wise labels. The labels consist of RGB images where white is the background, red is the inner pillar, green is the outer pillar, and blue is the tissue itself (Figure S2). The data was resized to the same dimensions before it was fed to the CNN. First, height and width are scaled to maintain the aspect ratio. Then padding is used to fill up space to the correct dimensions. The resized images have the dimension of 738 × 266 pixels.

#### 5.4.2 Training

A CNN was set up to segment the regions of the EHTs, using as input of the CNN a brightfield frame of an EHT video. Later, the mask matrices were generated by the shapes found using CNN. Then, the CNN is fed with frames of the EHT videos where the correct segmented output is known (labeled). We compared the output of the CNN with the known label by a cost function. Using the outcome of the comparison the CNN parameters were adjusted. We tested the precision of the CNN with a so‐called test‐set. The test‐set contains labeled frames that are new to the CNN, otherwise this will highly influence the outcome score. A score was given for precision by comparing the outputs of the CNN with the labels. We compared two CNN approaches for the segmentation of the EHT video frames, U‐net^40^ and Deeplabv3 with a ResNet‐101 backbone.^30^

The training of the U‐net model was done using the settings in Table 3. The input layer of the model was adjusted from a three‐layer RGB input to a one‐layer grayscale input since the input is in grayscale. For the training of the Deeplab with a ResNet‐101 backbone, we used the settings in Table 3. The input layer of the model is in three‐layer RGB format. Since the model uses the three layers further up in the model, the input layer cannot be changed directly. Therefore, the grayscale input was converted to an RGB image before it was fed to the model.

**TABLE 3:** Training settings for U‐net and Deeplabv3 with a ResNet‐101 backbone on the EHT dataset.

	U‐net	Deeplabv3 with a ResNet‐101 backbone
Loss function	Mean square error (MSE)	MSE
Optimizer	Adaptive momentum (Adam)	Adam
Learn rate	0.05	0.01
Epochs	200	200
Batch size	1	3
Input layers	1	3
Output layers	4	4

#### 5.4.3 Calculation the 3D cardiac tissue surface area

We calculated the surface area of the 3D cardiac tissues by calculating the surface of one pixel and multiplying it by the sum of all activated pixels in the segmented mask (Figure S2C), which contains all the pixels of the tissue. In Equation (8) the formula for calculating the surface area is shown.

(8) $$\text{Surface area}\;\left({\mathrm{mm}}^{2}\right)=a^{2}*\sum I_{\text{Surfacemask}},$$

where a is the conversion factor from pixels to millimeters.

#### 5.4.4 Segmentation by a deep learning model as input for template matching

The proposed template matching method (Section 5.2.1) requires as an input a template of the two pillars to track the displacement trajectory during tissue contraction. Previously, manual segmentations were described. However, we incorporated the results of the deep learning segmentation models as an input to the template matching and tracking algorithm. We performed a comprehensive comparison between manual segmentation, segmentation by U‐net, and segmentation by Deeplabv3 with a ResNet‐101 backbone. Using the mask of the outer pillar (Figure 3b), we made a square around it from the minimum and maximum bounds of its shape to create a template. To avoid any unwanted error by blobs in the segmentation, the two largest shapes in the segmentation were selected to initialize the templates.

#### 5.4.5 Visual inspection of tracking results

We checked the tracking and trajectory results of the algorithm by qualitatively scoring 81 videos with a grade from one to five. In the grading scale, one indicates that the tracking was not correct and as a consequence the trajectory is completely different from the expected one, while five indicates that the algorithm was able to track the center of the pillars precisely, resulting in computing a contraction wave as expected. We used the results overview of the user interface to evaluate each video.

### 5.5 Comparison of tracking accuracy between our method and MUSCLEMOTION

We used the simulation videos (described in Section 5.3) to compare the performance results achieved by MUSCLEMOTION and by our tracking algorithm. In the experiments, we considered increasing image noise levels from 1% to 25%, in order to simulate acquisition induced noise by the sensors and evaluate the algorithms in more challenging conditions. The results of both algorithms were compared to the ground truth contraction signal.

### 5.6 HPSC culture and generation of hPSC‐CMs

The experiments were done using a hiPSC (LUMC0020iCTRL‐06^/+^, female)^47^ line and a double reporter hESC (NKX2.5^EGFP/+^‐COUP‐TFII^mCherry/+^, female).^48^, ^49^ hiPSC and hESC were maintained as undifferentiated colonies in Essential 8 medium (Thermo Fisher, A1517001) on vitronectin (Thermo Fisher, A31804)‐coated 6‐well plates. The differentiation to hiPSC‐CMs and hESC‐CMs was induced as described previously.^50^ Briefly, 1 day before starting the differentiation, hiPSC and hESC were seeded separately at a density of 20–25 × 10^3^ cells per cm^2^ on Matrigel (83 μg protein/mL) (Corning, 354230) coated 6‐well plates in Essential 8 medium. After 24 h (day 0 (D0)), mesodermal differentiation was induced by addition of Activin‐A (20–30 ng/mL, Miltenyi 130–115‐010), BMP4 (20–30 ng/mL, R&D systems 314‐BP/CF), and Wnt activator CHIR99021 (1.5–2.25 μmol/L, Axon Medchem 1386) in BPEL medium.^51^ At day 3 (D3), BPEL containing WNT inhibitor XAV939 (5 μmol/L, R&D Systems 3748) was used to refresh the cells. On day 7 (D7) and 10 (D10) of differentiation, cells were refreshed with BPEL. Beating CMs at day 13 (D13) were metabolically selected with a lactate purification step of 4 days. This lactate purification medium consisted of our previously described maturation medium (MM)^50^ without glucose and with additional 5 mM of sodium dl‐lactate solution (60%, Sigma Aldrich, cat. no. L4263). At day 17 (D17), purified CMs were kept in the above‐described lactate purification medium with 4.5 mM of glucose for 3 more days, when cells were then dissociated with Triple 10× (ThermoFisher, A1217702) and cryopreserved (Figure S8A). Cells with at least 90% of green fluorescent protein (GFP) positive signal were used (Figure S9).

### 5.7 Cardiac fibroblast expansion

Primary human adult cardiac fibroblast (HCF) isolated from the ventricles of the adult heart were purchased from Promocell (C‐12375) and expanded according to the protocol.^52^ Briefly, a T175 cell culture flask (Greiner) was incubated (at 37 °C and 5% CO_2_) with 12 mL of FGM‐3 (Promocell, C‐23130) for 30 min. After thawing in a water bath at 37 °C, the cells were transferred from the cryovial to the pre‐incubated cell culture flask containing the FGM‐3 with an additional 18 mL of FGM‐3. Every 2 days, refreshments were done. After reaching a 70%–90% confluency in the flask, the cells were passaged; this process was repeated until reaching 11 passages. Then, the HCF were frozen at a final concentration of 150 × 10^3^ cells/ 0.5 mL in freezing medium. The freezing medium consists of 50% KOSR (Thermo Fisher, 10828028), 40% FGM‐3, 10% DMSO (Sigma‐Aldrich, D2650), and 0.5% Revitacell (Thermo Fisher, A2644501).

### 5.8 Generation of EHT

The EHTs were made as previously described by Ribeiro et al^35^ using the commercially available EHT platform provided by River Biomedics.^18^ Shortly, three tissues per well (12‐well plate format) were made using hiPSC‐CMs and hESC‐CMs, with a 3% HCF. First, the cells were thawed and resuspended in MM with 4.5 mM glucose and 5 mM sodium dl‐lactate. Then, 3% of HCF were mixed to a fixed amount of CMs (8 × 10^5^ cells for one well of the 12‐well plate) and each condition was resuspended to a final concentration of 16.8 × 10^6^ cells/mL. After that, cells were mixed with an extracellular matrix (ECM) mixture consisting of 2× MM medium, fibrinogen (final concentration 2 mg/mL, Sigma‐Aldrich F8630), Matrigel (final concentration 1 mg/mL), and aprotinin (final concentration 2.5 μg/mL, Sigma‐Aldrich, A1153). Finally, 0.6 U/mL of thrombin (Sigma, T7513) was added to the mix and 15 μL were used to make each one of the three tissues per well.^35^ The first refreshment was done after 24 h and after that every 2 or 3 days.

### 5.9 Generation of EHT using a commercial cell line

iCell CMs (11713 kit, R1105, female) were purchased from FUJIFILM Cellular Dynamics, Inc.^53^, ^54^ and used to make three tissues per well according to the previously described protocol.^35^ Briefly, iCells were thrown in the water bath and resuspended in iCell Cardiomyocytes Plating Medium. Then, 3% of HCF were mixed with a fixed amount of iCell (8 × 10^5^ cells for one well of the 12‐well plate) and resuspended to a final concentration of 16.8 × 10^6^cells/mL. Later, the cells were mixed with the ECM mixture (described in Section 5.8) and 15 μL of this mix was used to make each one of the three tissues per well. Then, 2 mL of iCell Cardiomyocytes Maintenance Medium was added to the well after the first 24 h and every refreshment was done every 2 or 3 days.

### 5.10 Mechanical characterization

The mechanical characterization of the River BioMedics commercial pillars was done according to Dostanić et al.^55^ Shortly, the force of 200 μN was applied on different positions along pillars' height using the micro‐force sensing probe of the FemtoTools Nanomechanical Testing System (FT‐NMT03). Elastic pillars were attached to a holder next to the sensing probe which was then positioned with nanometer precision at the predetermined height (Figure S7C). A force–displacement curve was obtained while applying force to the pillars using a flat silicon tip and measuring the displacement of the tip with piezo‐sensor. The slope of the force versus displacement curve represents the stiffness of the pillars.

### 5.11 Image‐based contractility measurement

The contractility of the EHTs in the commercial 12‐well plate was measured as previously described by Ribeiro et al.^35^ Briefly, the brightfield videos of EHTs were recorded using a Nikon ECLIPSE Ti2 inverted microscope (RRID:SCR_021068) under temperature and humidity control (37 °C and 5% CO_2_), using a high‐speed camera at 100 frames per second (fps) with 2× magnification. The output file was in ND2 format. Force of contraction was first measured at day 5 (D5) after tissue seeding and for a period of 30 days, every 5 days (day 10 (D10), 15 (D15), 20 (D20), 25 (D25), and 30 (D30)). During the force of contraction measurements, the EHTs were electrically stimulated using a custom‐made pacing device at 1 Hz (10 ms biphasic pulses, 4–5 V/cm) for 10 s (Figure S8B).

### 5.12 Compound testing

At day 30 (D30) the EHTs were assessed for a positive inotropic response to isoproterenol (Sigma, I5627). The test was conducted under temperature, humidity control (37 °C and 5% CO_2_), and field stimulation. The positive inotropic effect was determined with increasing concentrations of isoproterenol (0–10 μM). After 5 min of each dose administration, the tissues were recorded.

### 5.13 Statistics

Statistical analysis was performed using GraphPad Prism 8. Each experiment was performed three times, with CMs from independent differentiations. Per experiment, each set of three tissues (one well of a 12‐well plate) was considered as technical replicates. Differences between groups were assessed by two‐way ANOVA plus Tukey's post‐hoc test. Results are displayed as mean ± SD unless stated otherwise. Significance was attributed to comparisons with values of *p* < 0.05¥; *p* < 0.01†; *p* < 0.001‡; *p* < 0.0001#.

### 5.14 Python package

A python package was created for tracking with sub‐pixel precision to provide scientists with an easy‐to‐use library. This library does not contain the segmentation part since the segmentation relies on a specifically trained model, which will not work on slightly different images. Instead, the scientists can provide their own templates. The python package can be found on www.github.com/dkeekstra/sptemplatematching.

## AUTHOR CONTRIBUTIONS

**José Manuel Rivera Arbeláez:** Conceptualization (equal); data curation (equal); formal analysis (equal); investigation (equal); validation (equal); visualization (equal); writing – original draft (equal). **Danjel Keekstra:** Conceptualization (equal); data curation (equal); formal analysis (equal); investigation (lead); validation (equal); visualization (equal); writing – original draft (equal). **Carla Cofiño‐Fabres:** Validation (equal); writing – review and editing (equal). **Tom Boonen:** Validation (equal); writing – review and editing (equal). **Milica Dostanic:** Formal analysis (equal); validation (equal); writing – review and editing (equal). **Simone A. ten Den:** Validation (equal); writing – review and editing (equal). **Kim Vermeul:** Resources (equal); validation (equal); writing – review and editing (equal). **Massimo Mastrangeli:** Writing – review and editing (equal). **Albert van den Berg:** Funding acquisition (equal); project administration (equal); writing – review and editing (equal). **Loes I. Segerink:** Project administration (equal); resources (equal); supervision (equal); writing – review and editing (equal). **Marcelo C. Ribeiro:** Supervision (equal); writing – review and editing (equal). **Nicola Strisciuglio:** Conceptualization (equal); methodology (equal); resources (equal); supervision (equal); writing – review and editing (equal).

## FUNDING INFORMATION

This work was supported by the Netherlands Organ‐on‐Chip Initiative, an NWO Gravitation project (024.003.001) funded by the Ministry of Education, Culture and Science of the government of the Netherlands, TOP‐ZonMw grant (ZonMw, TOP‐00812‐98‐17061).

## CONFLICT OF INTEREST STATEMENT

R.P. is a cofounder of Pluriomics (Ncardia) and River BioMedics BV. M.C.R. is a cofounder of River BioMedics BV. A.v.d.B. is a scientific advisor of River BioMedics BV. The other authors report no conflicts.

### PEER REVIEW

The peer review history for this article is available at https://www.webofscience.com/api/gateway/wos/peer-review/10.1002/btm2.10513.

## Supporting information


**Figure S1.** Pillars of the 12‐well plate EHT platform provided by River BioMedics. (A) Variability in the shape of the pillars from a bottom view. (B) Three different brightfield image of hPSC‐derived EHT.
**Figure S2.** Segmented EHT frame. The labels consist of RGB images where white is the background, red is the inner pillar (A), green is the outer pillar (B), and blue is the tissue itself (C).
**Figure S3.** Simulation video of an EHT without noise.
**Figure S4.** Frame of simulation EHT videos. (A) Brightfield of an empty frame using 2× magnification. (B) Brightfield of an empty frame with two parts (right and left) of an EHT placed on top. (C) Frame of a simulation video with a 25% noise added on the EHT template.
**Figure S5.** Trajectory comparison. Comparison of output trajectory from nine EHT videos, MUSCLEMOTION versus template matching with sub‐pixel precision.
**Figure S6.** Tracking detection. Comparison of tracking detection between MUSCLEMOTION, template matching with sub‐pixel precision, and ground truth. Noise level from 1% to 25% was added to the video, to evaluate the performance of the algorithms.
**Figure S7.** Mechanical characterization of commercial pillars. (A) FemtoTools Nanomechanical Testing System (FT‐NMT03). (B) Analysis of experimental results to estimate the stiffness (and hence Young's modulus) of commercial pillars by analytical model (red) and experimental data (blue).
**Figure S8.** Flow chart of differentiation to CMs from hPSCs and experimental time line. (A) CM differentiation steps at day 0 (D0), 3 (D3), 7 (D7), 13 (D13), 17(D17), and 20 (D20). (B) Time line of contractile analysis carried every 5 days after tissue formation, specifically at day 5 (D5), 10 (D10), 15 (D15), 20 (D20), 25 (D25), and 30 (D30). Drug tests was performed at day 30 (D30). Created with BioRender.com.
**Figure S9.** Representative cardiomyocyte differentiation efficiency. Representative histogram plot of flow cytometry of differentiated COUP‐red (NKX2.5^eGFP/+^‐COUP‐TFII^mCherry/+^) CMs after lactate purification at day 20. Cardiomyocytes are quantified with the percentage of NKX2.5^EGFP+^ positive cells. Gray: negative control (NKX2.55^EGFP‐^) negative cells, green: NKX2.5^eGFP+^ positive cells.
**Table S1.** State‐of‐the‐art methods for contractile analysis using cantilevers as anchor points.

## Data Availability

The data that supports the findings of this study are available in the supplementary material of this article and in the link www.github.com/dkeekstra/sptemplatematching.

## References

[1] DiMasi JA , Grabowski HG , Hansen RW . Innovation in the pharmaceutical industry: new estimates of R&D costs. J Health Econ. 2016;47:20‐33. doi:10.1016/J.JHEALECO.2016.01.012 26928437

[2] Franzen N , van Harten WH , Retèl VP , Loskill P , van den Eijnden‐van Raaij J , IJzerman M . Impact of organ‐on‐a‐chip technology on pharmaceutical R&D costs. Drug Discov Today. 2019;24:1720‐1724. doi:10.1016/J.DRUDIS.2019.06.003 31185290

[3] Drug Discovery Market Size Worth Around US$ 161.76 Bn by 2030. n.d. Accessed July 29, 2022. https://www.precedenceresearch.com/drug-discovery-market

[4] Naci H , Carter AW , Mossialos E . Why the drug development pipeline is not delivering better medicines. BMJ. 2015;351:1‐4. doi:10.1136/bmj.h5542 26496934

[5] Savoji H , Mohammadi MH , Rafatian N , et al. Cardiovascular disease models: a game changing paradigm in drug discovery and screening. Biomaterials. 2019;198:3‐26. doi:10.1016/J.BIOMATERIALS.2018.09.036 30343824 PMC6397087

[6] Pang L , Sager P , Yang X , et al. Workshop report. Circ Res. 2019;125:855‐867. doi:10.1161/CIRCRESAHA.119.315378 31600125 PMC6788760

[7] Shah RR . Can pharmacogenetics help rescue drugs withdrawn from the market? Pharmacogenomics. 2006;7:889‐908. doi:10.2217/14622416.7.6.889 16981848

[8] Laverty HG , Benson C , Cartwright EJ , et al. How can we improve our understanding of cardiovascular safety liabilities to develop safer medicines? Br J Pharmacol. 2011;163:675‐693. doi:10.1111/J.1476-5381.2011.01255.X 21306581 PMC3111672

[9] Mercola M , Colas A , Willems E . iPSCs in cardiovascular drug discovery. Circ Res. 2013;112:534‐548. doi:10.1161/CIRCRESAHA.111.250266 23371902 PMC3706265

[10] Burridge PW , Matsa E , Shukla P , et al. Chemically defined generation of human cardiomyocytes. Nat Methods. 2014;11:855‐860. doi:10.1038/NMETH.2999 24930130 PMC4169698

[11] Passier R , Orlova V , Mummery C . Complex tissue and disease modeling using hiPSCs. Cell Stem Cell. 2016;18:309‐321. doi:10.1016/J.STEM.2016.02.011 26942851

[12] Mannhardt I , Saleem U , Mosqueira D , et al. Comparison of 10 control hPSC lines for drug screening in an engineered heart tissue format. Stem Cell Rep. 2020;15:983‐998. doi:10.1016/j.stemcr.2020.09.002 PMC7561618 33053362

[13] Zhao Y , Rafatian N , Wang EY , et al. Towards chamber specific heart‐on‐a‐chip for drug testing applications. Adv Drug Deliv Rev. 2020;165–166:60‐76. doi:10.1016/j.addr.2019.12.002 PMC7338250 31917972

[14] Vulto P , Joore J . Adoption of organ‐on‐chip platforms by the pharmaceutical industry. Nat Rev Drug Discov. 2021;20:961–962. 10.1038/s41573-021-00323-0 34646035

[15] Feric NT , Pallotta I , Singh R , et al. Engineered cardiac tissues generated in the biowire™ II: a platform for human‐based drug discovery. Toxicol Sci. 2019;172:89‐97. doi:10.1093/toxsci/kfz168 31385592 PMC6813749

[16] Naito H , Melnychenko I , Didié M , et al. Optimizing engineered heart tissue for therapeutic applications as surrogate heart muscle. Circulation. 2006;114:172‐178. doi:10.1161/CIRCULATIONAHA.105.001560 16820649

[17] Hansen A , Eder A , Bönstrup M , et al. Development of a drug screening platform based on engineered heart tissue. Circ Res. 2010;107:35‐44. doi:10.1161/CIRCRESAHA.109.211458 20448218

[18] 3D Cardiac Strip – River Biomedics. n.d. Accessed January 13, 2022. https://riverbiomedics.com/index.php/portfolio/cardiac-strip/

[19] Ronaldson‐Bouchard K , Ma SP , Yeager K , et al. Advanced maturation of human cardiac tissue grown from pluripotent stem cells. Nature. 2018;556(7700):239‐243. doi:10.1038/s41586-018-0016-3 29618819 PMC5895513

[20] Mills RJ , Parker BL , Quaife‐Ryan GA , et al. Drug screening in human PSC‐cardiac organoids identifies pro‐proliferative compounds acting via the mevalonate pathway. Cell Stem Cell. 2019;24:895‐907.e6. doi:10.1016/j.stem.2019.03.009 30930147

[21] Rivera‐Arbeláez JM , Cofiño‐Fabres C , Schwach V , et al. Contractility analysis of human engineered 3D heart tissues by an automatic tracking technique using a standalone application. PLoS One. 2022;17:e0266834. doi:10.1371/JOURNAL.PONE.0266834 35421132 PMC9009597

[22] Sala L , van Meer BJ , Tertoolen LT , et al. MUSCLEMOTION: a versatile open software tool to quantify cardiomyocyte and cardiac muscle contraction in vitro and in vivo. Circ Res. 2018;122(3):e5‐e16. doi:10.1161/CIRCRESAHA.117.312067 29282212 PMC5805275

[23] Wen C , Miura T , Voleti V , et al. 3DeeCellTracker, a deep learning‐based pipeline for segmenting and tracking cells in 3D time lapse images. eLife. 2021;10:1‐37. 10.7554/eLife.59187 PMC8009680 33781383

[24] Ghanbari M . The cross‐search algorithm for motion estimation. IEEE Trans Commun. 1990;38:950‐953. doi:10.1109/26.57512

[25] Hayakawa T , Kunihiro T , Dowaki S , et al. Noninvasive evaluation of contractile behavior of cardiomyocyte monolayers based on motion vector analysis. Tissue Eng Part C Methods. 2012;18:21‐32. doi:10.1089/TEN.TEC.2011.0273 21851323

[26] Thomas LSV , Gehrig J . Multi‐template matching: a versatile tool for object‐localization in microscopy images. BMC Bioinform. 2020;21:1‐8. doi:10.1186/S12859-020-3363-7/TABLES/1 PMC7003318 32024462

[27] Yu S , Xiao J , Zhang B , Lim G , Zhao Y . Fast pixel‐matching for video object segmentation. Proc IEEE/CVF Conf Comput Vis Pattern Recognit. 2020;10:791‐799.

[28] Maqsood K , Ali A , Ilyas SU , et al. Multi‐objective optimization of thermophysical properties of multiwalled carbon nanotubes based nanofluids. Chemosphere. 2022;286:131690. doi:10.1016/j.chemosphere.2021.131690 34352553

[29] Bradski G , Kaehler A . Learning OpenCV 3: Computer Vision in C++ with the OpenCV Library. O'Reilly Media, Inc; 2013. ISBN:9781491937990.

[30] Chen L‐C , Papandreou G , Schroff F , Adam H . Rethinking Atrous Convolution for Semantic Image Segmentation. Cornell University; 2017.

[31] Leonard A , Bertero A , Powers JD , et al. Afterload promotes maturation of human induced pluripotent stem cell derived cardiomyocytes in engineered heart tissues. J Mol Cell Cardiol. 2018;118:147‐158. doi:10.1016/J.YJMCC.2018.03.016 29604261 PMC5940558

[32] Mills RJ , Titmarsh DM , Koenig X , et al. Functional screening in human cardiac organoids reveals a metabolic mechanism for cardiomyocyte cell cycle arrest. Proc Natl Acad Sci U S A. 2017;114:E8372‐E8381. doi:10.1073/PNAS.1707316114 28916735 PMC5635889

[33] Sniadecki NJ , Chen CS . Microfabricated silicone elastomeric post arrays for measuring traction forces of adherent cells. Methods Cell Biol. 2007;83:313‐328. doi:10.1016/S0091-679X(07)83013-5 17613314

[34] Serrao GW , Turnbull IC , Ancukiewicz D , et al. Myocyte‐depleted engineered cardiac tissues support therapeutic potential of mesenchymal stem cells. Tissue Eng Part A. 2012;18:1322‐1333. doi:10.1089/TEN.TEA.2011.0278 22500611 PMC3397121

[35] Ribeiro MC , Rivera‐Arbeláez JM , Cofiño‐Fabres C , et al. A new versatile platform for assessment of improved cardiac performance in human‐engineered heart tissues. J Pers Med. 2022;12:214. doi:10.3390/JPM12020214 35207702 PMC8877418

[36] Mills RJ , Hudson JE . Bioengineering adult human heart tissue: how close are we? APL Bioeng. 2019;3:010901. doi:10.1063/1.5070106 31069330 PMC6481734

[37] Qu Y , Feric N , Pallotta I , Singh R , Sobbi R , Vargas HM . Inotropic assessment in engineered 3D cardiac tissues using human induced pluripotent stem cell‐derived cardiomyocytes in the Biowire^TM^ II platform. J Pharmacol Toxicol Methods. 2020;105:106886. doi:10.1016/j.vascn.2020.106886 32629159

[38] Chen L‐C , Papandreou G , Schroff F , Adam H . Rethinking atrous convolution for semantic image segmentation. arXiv. 2017;1706.05587v3. doi:10.48550/arxiv.1706.05587

[39] Van Valen DA , Kudo T , Lane KM , et al. Deep learning automates the quantitative analysis of individual cells in live‐cell imaging experiments. PLoS Comput Biol. 2016;12:e1005177. doi:10.1371/JOURNAL.PCBI.1005177 27814364 PMC5096676

[40] Ronneberger O , Fischer P , Brox T . U‐net: convolutional networks for biomedical image segmentation. Lect Notes Comput Sci (Including Subser Lect Notes Artif Intell Lect Notes Bioinformatics). 2015;9351:234‐241. doi:10.48550/arxiv.1505.04597

[41] Mannhardt I , Breckwoldt K , Letuffe‐Brenière D , et al. Human engineered heart tissue: analysis of contractile force. Stem Cell Rep. 2016;7:29‐42. doi:10.1016/j.stemcr.2016.04.011 PMC4944531 27211213

[42] Nunes SS , Miklas JW , Liu J , et al. Biowire: a platform for maturation of human pluripotent stem cell‐derived cardiomyocytes. Nat Methods. 2013;10:781‐787. doi:10.1038/nmeth.2524 23793239 PMC4071061

[43] Ribeiro MC , Slaats RH , Schwach V , et al. A cardiomyocyte show of force: a fluorescent alpha‐actinin reporter line sheds light on human cardiomyocyte contractility versus substrate stiffness. J Mol Cell Cardiol. 2020;141:54‐64. doi:10.1016/j.yjmcc.2020.03.008 32205183

[44] Eder A , Vollert I , Hansen A , Eschenhagen T . Human engineered heart tissue as a model system for drug testing. Adv Drug Deliv Rev. 2016;96:214‐224. doi:10.1016/j.addr.2015.05.010 26026976

[45] Press WH , Teukolsky SA , Vetterling WT , Flannery BP . The Art of Scientific Computing. 2nd ed. Cambridge University Press; 1988.

[46] Singer S , Nelder J . Nelder‐Mead Algorithm. Scholarpedia. 2009;4:2928. doi:10.4249/SCHOLARPEDIA.2928

[47] LUMCi028‐A · Cell Line · hPSCreg. n.d. Accessed August 5, 2022. https://hpscreg.eu/cell-line/LUMCi028-A

[48] Schwach V , Verkerk AO , Mol M , et al. A COUP‐TFII human embryonic stem cell reporter line to identify and select atrial cardiomyocytes. Stem Cell Rep. 2017;9:1765‐1779. doi:10.1016/j.stemcr.2017.10.024 PMC5785710 29173897

[49] ESIBIe003‐A · Cell Line · hPSCreg. n.d. Accessed February 13, 2023. https://hpscreg.eu/cell-line/ESIBIe003-A

[50] Birket MJ , Ribeiro MC , Kosmidis G , et al. Contractile defect caused by mutation in MYBPC3 revealed under conditions optimized for human PSC‐cardiomyocyte function. Cell Rep. 2015;13:733‐745. doi:10.1016/j.celrep.2015.09.025 26489474 PMC4644234

[51] Ng ES , Davis R , Stanley EG , Elefanty AG . A protocol describing the use of a recombinant protein‐based, animal product‐free medium (APEL) for human embryonic stem cell differentiation as spin embryoid bodies. Nat Protoc. 2008;3:768‐776. doi:10.1038/nprot.2008.42 18451785

[52] PromoCell . Instruction Manual. n.d.

[53] iCell Cardiomyocytes, 11713, GCMC11713 | FujiFilm CDI n.d. Accessed January 27, 2022. https://www.fujifilmcdi.com/icell-cardiomyocytes-11713-gcmc11713

[54] CDIi004‐A · Cell Line · hPSCreg. n.d. Accessed February 13, 2023. https://hpscreg.eu/cell-line/CDIi004-A

[55] Dostanić M , Windt LM , Stein JM , et al. A miniaturized EHT platform for accurate measurements of tissue contractile properties. J Microelectromech Syst. 2020;29:881‐887. doi:10.1109/JMEMS.2020.3011196

