# Supplementary material for: Automated assessment of human engineered heart tissues using deep learning and template matching for segmentation and tracking
Source: Bioeng Transl Med. 2023 Apr 18;8(3):e10513. doi: 10.1002/btm2.10513 (PMC10189437; doi:10.1002/btm2.10513)
Supplement: Supplementary file 1 — Figure S1. Pillars of the 12‐well plate EHT platform provided by River BioMedics. (A) Variability in the shape of the pillars from a bottom view. (B) Three different brightfield image of hPSC‐derived EHT. Figure S2. Segmented EHT frame. The labels consist of RGB images where white is the background, red is the inner pillar (A), green is the outer pillar (B), and blue is the tissue itself (C). Figure S3. Simulation video of an EHT without noise. Figure S4. Frame of simulation EHT videos. (A) Brightfield of an empty frame using 2× magnification. (B) Brightfield of an empty frame with two parts (right and left) of an EHT placed on top. (C) Frame of a simulation video with a 25% noise added on the EHT template. Figure S5. Trajectory comparison. Comparison of output trajectory from nine EHT videos, MUSCLEMOTION versus template matching with sub‐pixel precision. Figure S6. Tracking detection. Comparison of tracking detection between MUSCLEMOTION, template matching with sub‐pixel precision, and ground truth. Noise level from 1% to 25% was added to the video, to evaluate the performance of the algorithms. Figure S7. Mechanical characterization of commercial pillars. (A) FemtoTools Nanomechanical Testing System (FT‐NMT03). (B) Analysis of experimental results to estimate the stiffness (and hence Young's modulus) of commercial pillars by analytical model (red) and experimental data (blue). Figure S8. Flow chart of differentiation to CMs from hPSCs and experimental time line. (A) CM differentiation steps at day 0 (D0), 3 (D3), 7 (D7), 13 (D13), 17(D17), and 20 (D20). (B) Time line of contractile analysis carried every 5 days after tissue formation, specifically at day 5 (D5), 10 (D10), 15 (D15), 20 (D20), 25 (D25), and 30 (D30). Drug tests was performed at day 30 (D30). Created with BioRender.com. Figure S9. Representative cardiomyocyte differentiation efficiency. Representative histogram plot of flow cytometry of differentiated COUP‐red (NKX2.5eGFP/+‐COUP‐TFIImCherry/+) CMs a [file BTM2-8-e10513-s001.docx]

# Supplementary material


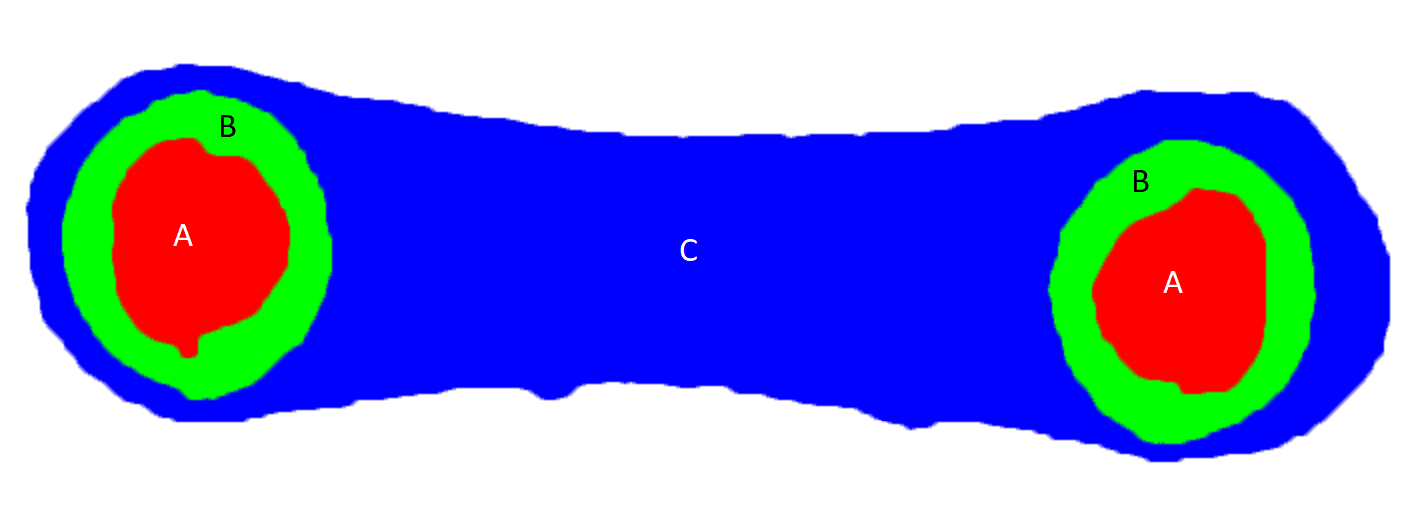


Figure S2. Segmented EHT frame. The labels consist of RGB images where white is the background, red is the inner pillar (A), green is the outer pillar (B), and blue is the tissue itself (C).


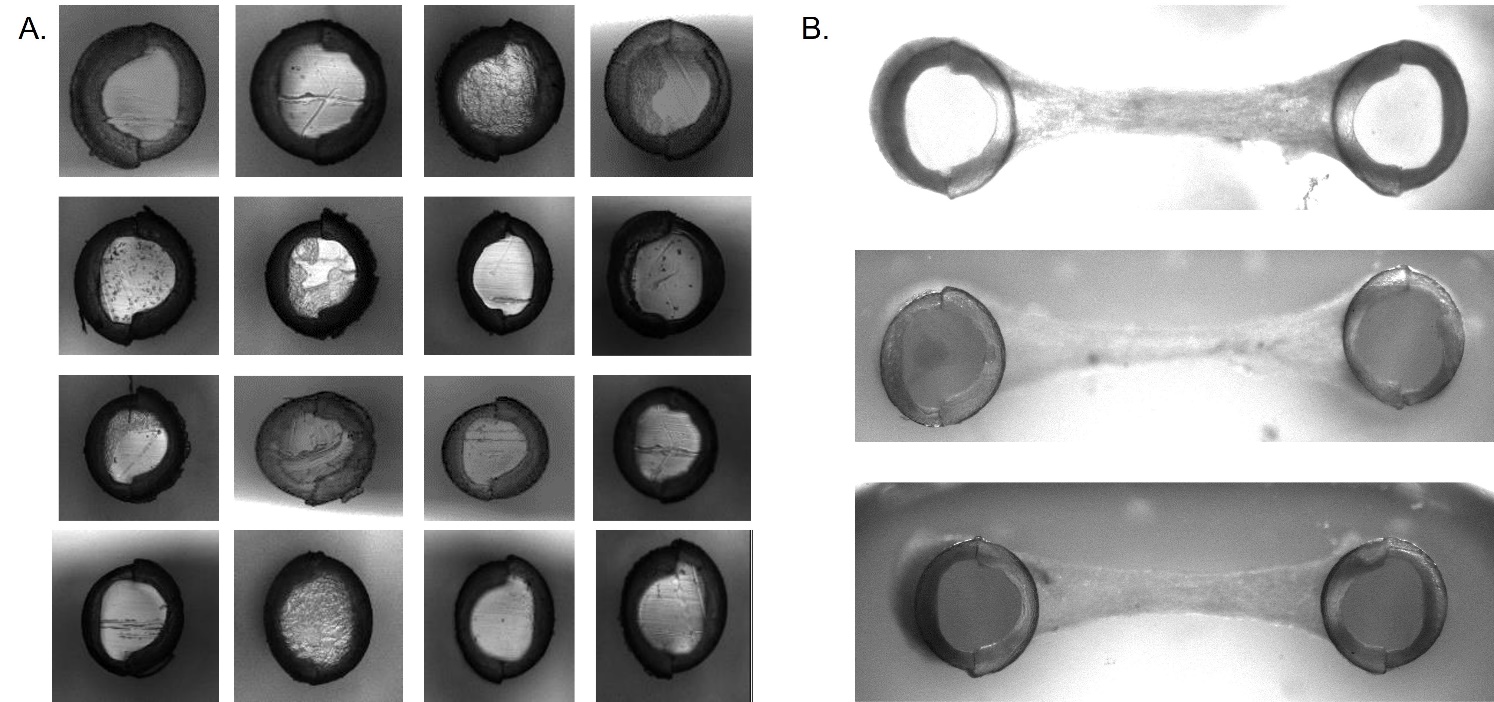


Figure S1. Pillars of the 12 well-plate EHT platform provided by River BioMedics. (A) Variability in the shape of the pillars from a bottom view. (B) Three different brightfield image of hPSC-derived EHT.


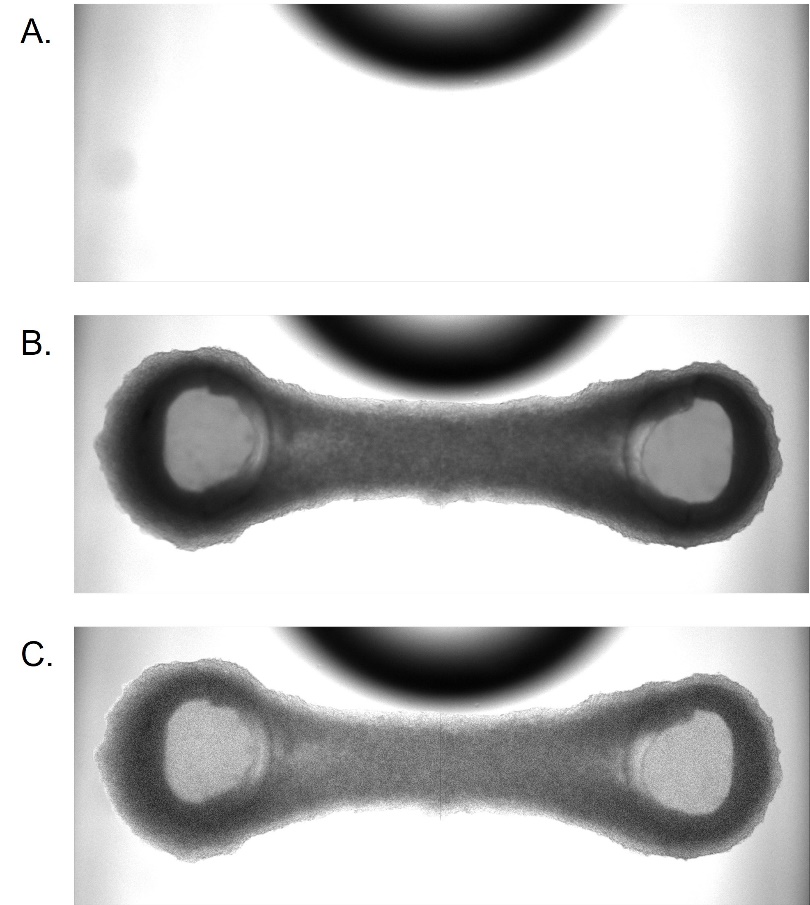


Figure S4. Frame of simulation EHT videos. (A) Brightfield of an empty frame using 2X magnification. (B) Brightfield of an empty frame with two parts (right and left) of an EHT placed on top. (C) Frame of a simulation video with a 25% noise added on the EHT template.


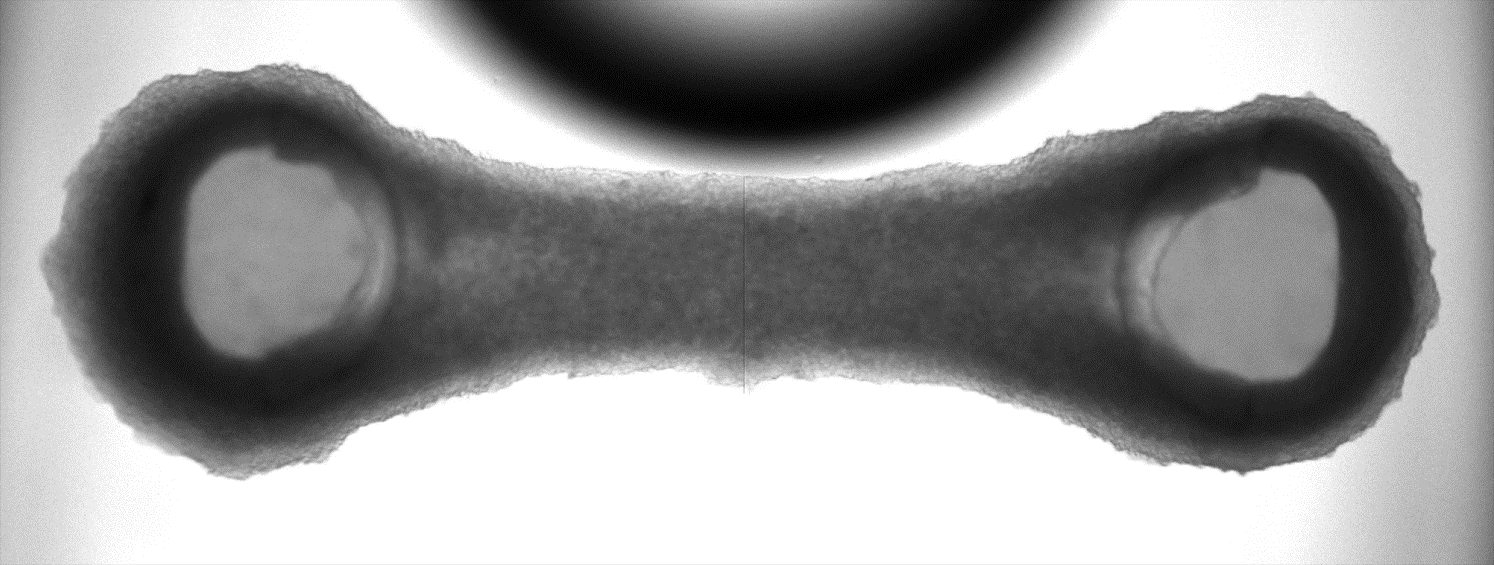


Figure S3. Simulation video of an EHT without noise.


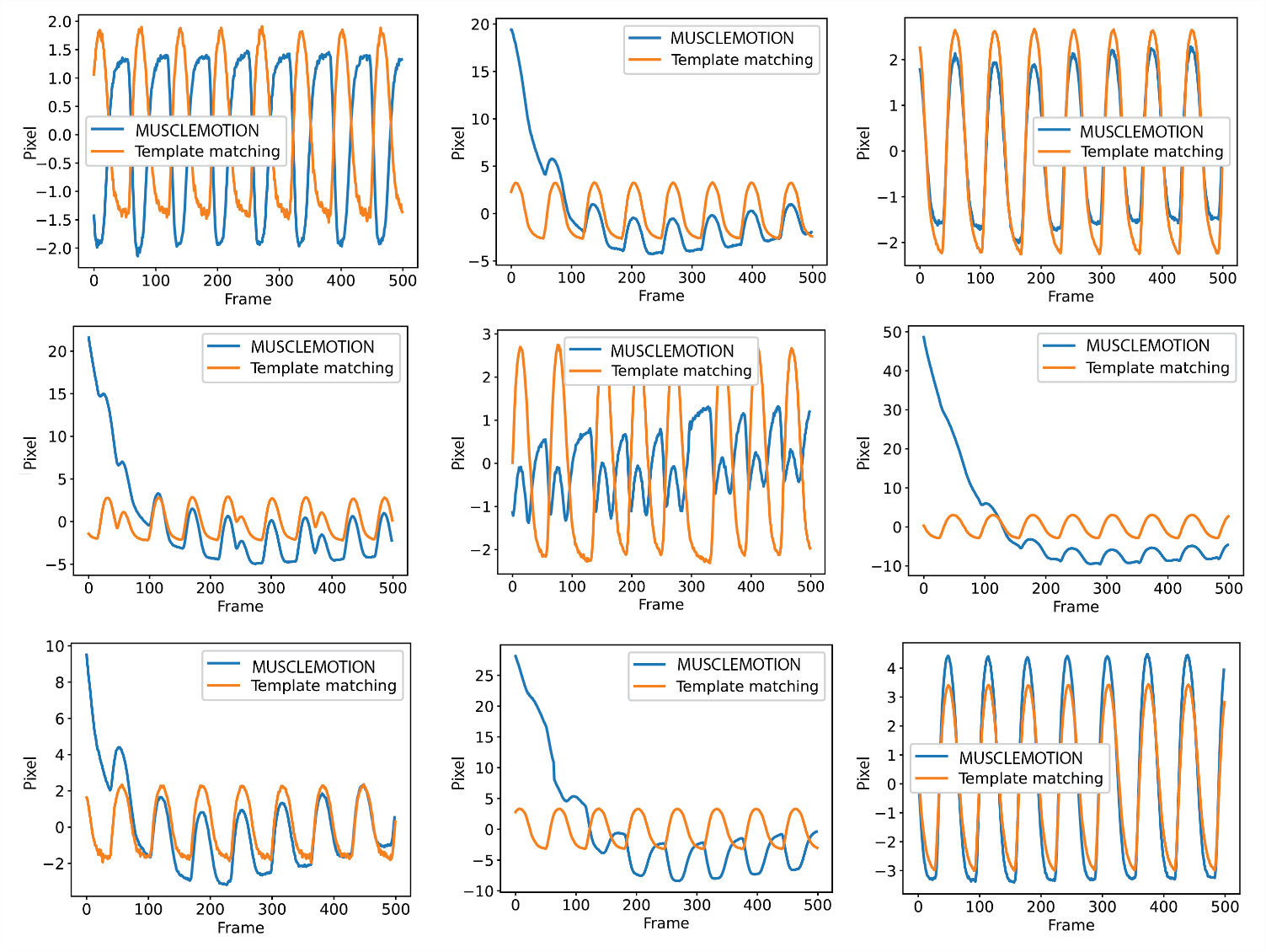


**Figure S5**. **Trajectory comparison**. Comparison of output trajectory from nine EHT videos, MUSCLEMOTION versus template matching with sub-pixel precision.


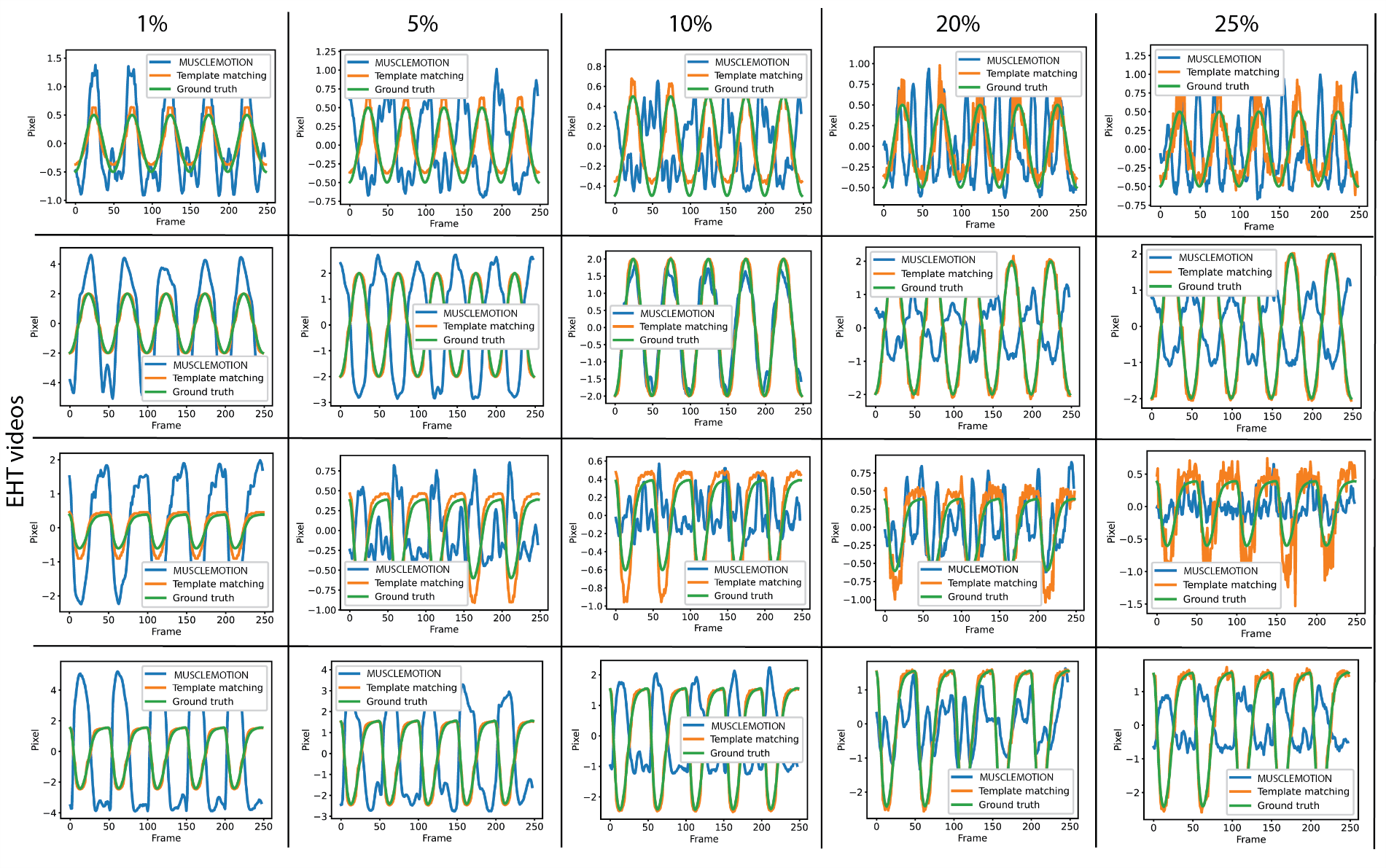


**Figure S6.** **Tracking detection**. Comparison of tracking detection between MUSCLEMOTION, template matching with sub-pixel precision and ground truth. Noise level from 1% to 25% was added to the video, to evaluate the performance of the algorithms.

Figure S7. Mechanical characterization of commercial pillars. (A) FemtoTools Nanomechanical Testing System (FT-NMT03). (B) Analysis of experimental results to estimate the stiffness (and hence Young’s modulus) of commercial pillars by analytical model (red) and experimental data (blue).


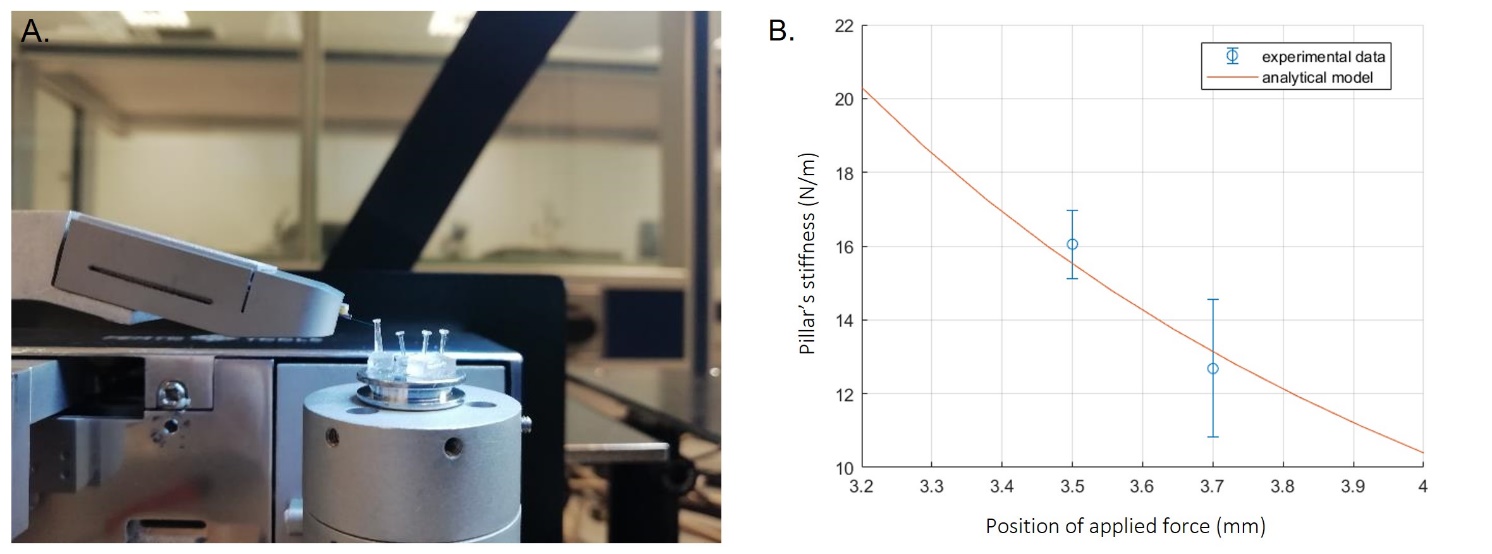

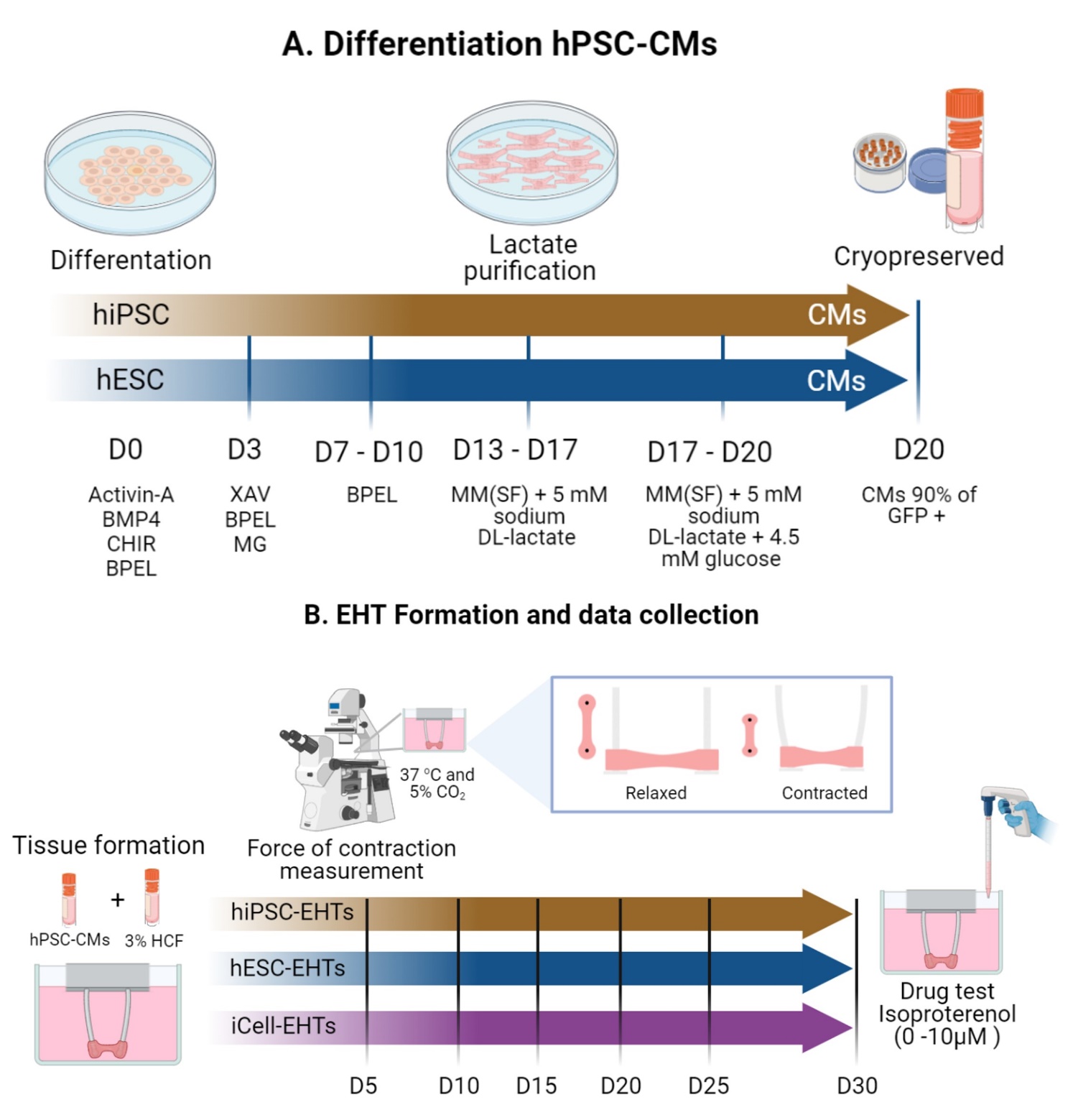


Figure S8. Flow chart of differentiation to CMs from hPSCs and experimental time line. (A) CM differentiation steps at day 0 (D0), 3 (D3), 7 (D7), 13 (D13), 17(D17) and 20 (D20). (B) Time line of contractile analysis carried every five days after tissue formation, specifically at day 5(D5), 10(D10), 15(D15), 20(D20), 25(D25), and 30(D30). Drug tests was performed at day 30 (D30). Created with BioRender.com.


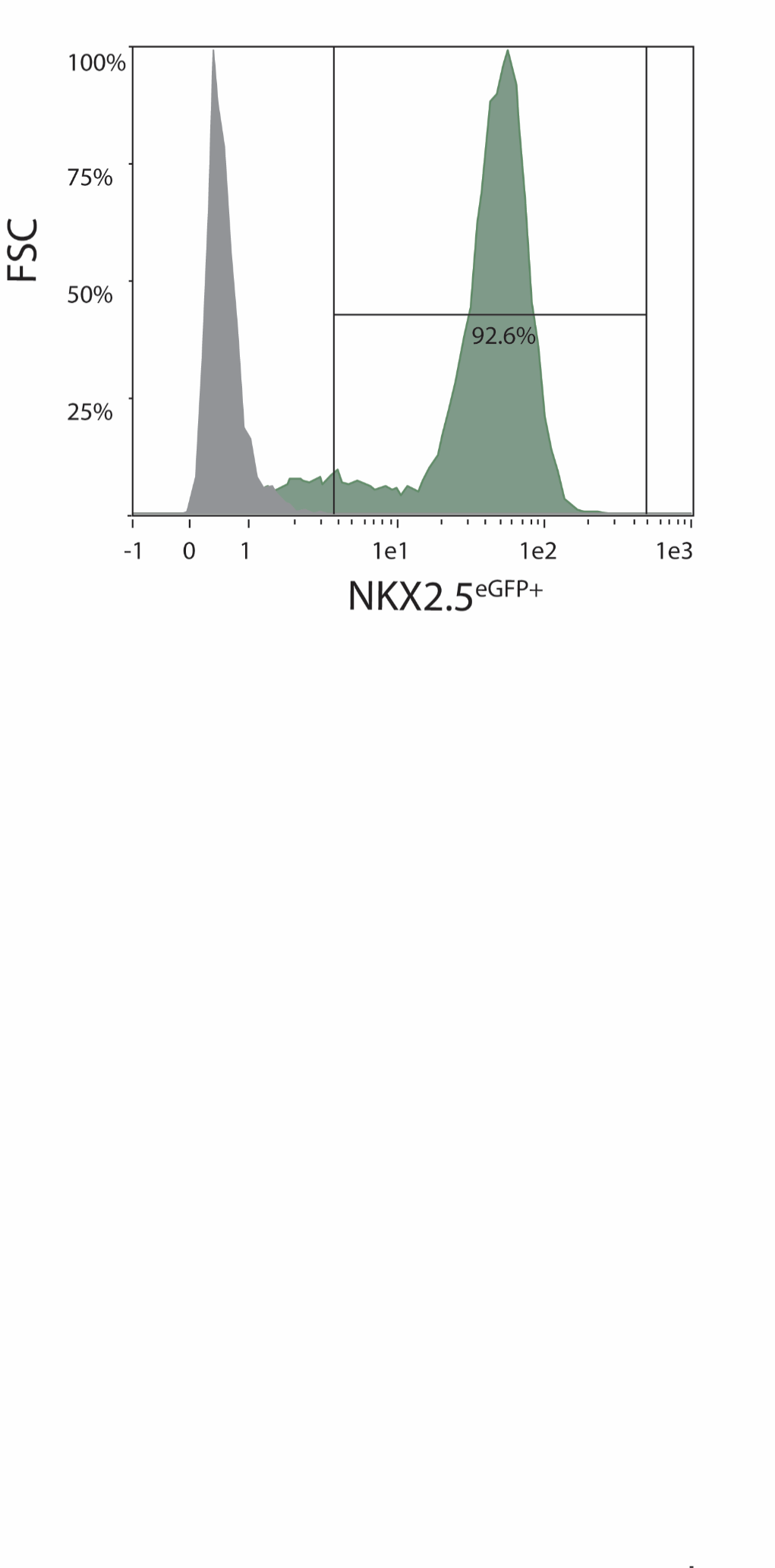


**Figure S9. Representative cardiomyocyte differentiation efficiency.** Representative histogram plot of flow cytometry of differentiated COUP-red (NKX2.5^eGFP/+^-COUP-TFII^mCherry/+^) CMs after lactate purification at day 20. Cardiomyocytes are quantified with the percentage of NKX2.5^eGFP+^) positive cells. Grey: negative control (NKX2.55^Egfp-^) negative cells), green: NKX2.5^eGFP+^) positive cells.

**Table S1.** State-of-the-art methods for contractile analysis using cantilevers as anchor points

| **Paper** | **Approach** | **Format** | **Type of software** | **Output** | **Additional information** |
| --- | --- | --- | --- | --- | --- |
| Hansen et al [35]  PMID: 20448218 | Uses of figure recognition of the contracting muscle strip at top and bottom ends, in a fully automated manner. Records of experiments are automatically generated with two levels of quality control: pictures are taken at the beginning and the end of each measurement and blue squares indicate the positions on both ends of the muscle stripe | Bright field | Customized software package by Consulting Team Machine Vision (Pforzheim, Germany) | -Frequency  -Average force  -Fractional shortening, contraction – and relaxation time (T1, T2, respectively). | Custom microscope settings required. Setup is limited to defining the x, y, z coordinates of the camera for each tissue. |
| Serrao et al [34]  PMID:22500611 | The post position as a function of time is documented for each contraction, by analyzing the change in distance between the post centroids | Bright field | Custom MATLAB routines (MathWorks, Natick, MA) | -Frequency  -Force | Force per CSA and force per myocyte is calculated in a second step. |
| Sniadecki et al [33]  PMID: 17613314 | The centroids of the fluorescent micropost are calculated by performing a localized thresholding algorithm. This routine is repeated for both top and bottom images and generates respective matrices of centroid positions. Traction force is calculated as the difference in positions between the top and bottom images. | Fluorescent image | Custom MATLAB image-processing toolbox (MathWorks, Natick, MA) | -Cell-generated forces | Uses of high-magnification objectives (60x) with oil to acquire top and bottom images of the microposts is required. |
| Sala et al[22] PMID: 29282212 | The principle of MUSCLEMOTION is the assessment of contraction using an intuitive approach quantifying absolute changes in pixel intensity between a reference frame and the frame of interest. | Bright field | ImageJ Macro-open source software | -Contractile force  -Contractile velocity  -Time- to-peak and relaxation time |  |
| Mills et al [32]  PMID: 28916735 | The poles movement are tracked by using vision PointTracker after converting the stacked .tiff image files to .avi movie files. | Bright field | Custom MATLAB routines R2013a (MathWorks, Natick, MA) using vision.PointTracker | -Contractile force  -Contractile velocity  -Time- to-peak and relaxation time  -Force-time figure  -Batch data to an Excel (Microsoft) spreadsheet |  |
| Leonard et al [46]  PMID:29604261 | The centroids of the flexible post is track relative to the centroid of the rigid post by thresholding the images using a custom Matlab program. | Bright field | Custom MATLAB image-processing toolbox (MathWorks, Natick, MA) | -Twitch force  -Velocity  -Power  -Work |  |
| Ronaldson-Bouchard et al [62]  PMID: 29618819 | The video frames are inverted and an automated intensity threshold is used to identify cell location in the video frame. The baseline timepoint in the video corresponding to a relaxed tissue state is selected and absolute differences in cell area from the baseline frame are then calculated to create a time course of cell area dynamics as a function of time | Bright field | Custom MATLAB routines (MathWorks, Natick, MA) | -Beat period  -Beat frequencies  -Contraction amplitude  -Relaxation times (R90) | Live cell, bright field videos are acquired at rates of up to 150 frames per second using a Pike F-032b (Allied Vision Technologies) camera controlled with custom SPLASSH software |
